# The effects of soil phosphorus content on plant microbiota are driven by the plant phosphate starvation response

**DOI:** 10.1371/journal.pbio.3000534

**Published:** 2019-11-13

**Authors:** Omri M. Finkel, Isai Salas-González, Gabriel Castrillo, Stijn Spaepen, Theresa F. Law, Paulo José Pereira Lima Teixeira, Corbin D. Jones, Jeffery L. Dangl

**Affiliations:** 1 Department of Biology, University of North Carolina at Chapel Hill, Chapel Hill, North Carolina, United States of America; 2 Howard Hughes Medical Institute, University of North Carolina at Chapel Hill, Chapel Hill, North Carolina, United States of America; 3 Curriculum in Bioinformatics and Computational Biology, University of North Carolina at Chapel Hill, Chapel Hill, North Carolina, United States of America; 4 Department Plant Microbe Interactions, Max Planck Institute for Plant Breeding Research, Köln, Germany; 5 Department of Genetics, University of North Carolina at Chapel Hill, Chapel Hill, North Carolina, United States of America; 6 Lineberger Comprehensive Cancer Center, University of North Carolina at Chapel Hill, Chapel Hill, North Carolina, United States of America; 7 Carolina Center for Genome Sciences, University of North Carolina at Chapel Hill, Chapel Hill, North Carolina, United States of America; 8 Curriculum in Genetics and Molecular Biology, University of North Carolina at Chapel Hill, Chapel Hill, North Carolina, United States of America; 9 Department of Microbiology and Immunology, University of North Carolina at Chapel Hill, Chapel Hill, North Carolina, United States of America; University of California Davis, UNITED STATES

## Abstract

Phosphate starvation response (PSR) in nonmycorrhizal plants comprises transcriptional reprogramming resulting in severe physiological changes to the roots and shoots and repression of plant immunity. Thus, plant-colonizing microorganisms—the plant microbiota—are exposed to direct influence by the soil’s phosphorus (P) content itself as well as to the indirect effects of soil P on the microbial niches shaped by the plant. The individual contribution of these factors to plant microbiota assembly remains unknown. To disentangle these direct and indirect effects, we planted PSR-deficient *Arabidopsis* mutants in a long-term managed soil P gradient and compared the composition of their shoot and root microbiota to wild-type plants across different P concentrations. PSR-deficiency had a larger effect on the composition of both bacterial and fungal plant-associated microbiota than soil P concentrations in both roots and shoots.

To dissect plant–microbe interactions under variable P conditions, we conducted a microbiota reconstitution experiment. Using a 185-member bacterial synthetic community (SynCom) across a wide P concentration gradient in an agar matrix, we demonstrated a shift in the effect of bacteria on the plant from a neutral or positive interaction to a negative one, as measured by rosette size. This phenotypic shift was accompanied by changes in microbiota composition: the genus *Burkholderia* was specifically enriched in plant tissue under P starvation. Through a community drop-out experiment, we demonstrated that in the absence of *Burkholderia* from the SynCom, plant shoots accumulated higher ortophosphate (Pi) levels than shoots colonized with the full SynCom but only under Pi starvation conditions. Therefore, Pi-stressed plants are susceptible to colonization by latent opportunistic competitors found within their microbiome, thus exacerbating the plant’s Pi starvation.

## Abstract 2

This study shows that plants' response to phosphate starvation has more influence on plant microbiota than does the phosphate concentration itself; these changes are not necessarily beneficial for the plant, and can even exacerbate the phosphate starvation.

## Introduction

Plants provide the primary energy source for terrestrial heterotrophs, most of which are microbial. The interaction of these microbial heterotrophs with plants ranges between the extremes of mutualistic symbiosis [1] and pathogenesis [2,3]. However, the vast majority of plant-associated microbial diversity, the plant microbiota, lies between these 2 extremes, inducing more subtle, context-dependent effects on plant health [4–6]. The microbiota consumes plant photosynthate [7–9], and it provides benefits via protection from pathogens [10–14] or abiotic stress [15,16] or by increasing nutrient bioavailability [4,17,18].

The plant microbiota is derived from the microbial community composition in soil [19–21], which is governed by its own set of ecological processes [22]. Correlations with soil microbial diversity, and by derivation, with plant microbiota composition and diversity, were observed for soil abiotic factors, such as pH [22–25], drought [25–30], and nutrient concentrations [22,25,31–35]. Soil nutrient concentrations, in particular orthophosphate (Pi)—the only form of phosphorus (P) that can be taken up by plants—produce comparatively modest changes in microbial community composition [35,36]. Nevertheless, available soil Pi concentrations influences where a plant–microbe interaction lies along the mutualism–pathogenicity continuum [17].

Nonmycorrhizal plants respond to phosphate limitation by employing a range of PSR mechanisms. These manifest as severe physiological and morphological changes to the root and shoot, such as lateral root growth prioritization, depletion of shoot Pi stores [37], and changes to root exudate profiles [38,39]; these changes can potentially affect both root and shoot microbiota. In *Arabidopsis*, most of the transcriptional PSR driving these physiological responses is controlled by the 2 partially redundant transcription factors PHOSPHATE STARVATION RESPONSE 1 (PHR1) and PHR1-LIKE (PHL1) [40]. As a result, the double mutant *phr1 phl1* has an impaired PSR and accumulates a low level of Pi. Pi transport into roots relies on the PHOSPHATE TRANSPORTER TRAFFIC FACILITATOR1 (PHF1) gene, which is required for membrane localization of high-affinity phosphate transporters [41]. In axenic conditions, *phf1* mutants constitutively express PSR and accumulate low levels of Pi [41]. In addition to inducing physiological changes, the plant’s response to its nutrient status is also linked to its immune system. PHR1 negatively regulates components of the plant immune system, which can lead to enhanced pathogen susceptibility but also to the alteration of the plant’s microbiota under phosphate starvation [4]. *Arabidopsis* microbiota are altered in *phr1 phl1* and *phf1* mutants [4,36] in experiments using both natural and synthetic microbial communities [4].

Here, we examined (i) the effect of soil P content on plant microbiota composition, (ii) how PSR modulates the plant microbiota, and (iii) the interplay between PSR and soil P content in shaping the plant microbiota composition. We used a combination of greenhouse experiments with differentially P-fertilized soils, *Arabidopsis* PSR mutants and laboratory microcosms utilizing tractable synthetic bacterial communities. Using PSR mutants planted in P-amended soil, we demonstrate that the plant PSR regulators have a profound effect on the composition of root and shoot microbiota, overshadowing the effect of the soil P content. We constructed an ecologically tractable system utilizing a complex bacterial synthetic community (SynCom) as a model of the plant root microbiome and used this system to study the interactions between microbiota assembly and abiotic stress. We demonstrate deterministic responses of the SynCom members to changes in Pi concentrations, and we identify Pi-dependent shifts in community composition along the mutualist–pathogen continuum.

## Results

### PSR is activated in soil

To better understand the effect of PSR genes on the plant microbiome under both Pi-limiting and Pi-replete conditions, we investigated how microbiota adapted to varying soil P levels interact with the plant’s PSR. We grew wild-type (wt) *Arabidopsis* and the PSR mutants *phf1* and *phr1 phl1* in soils collected from the “Halle long-term soil fertilization experiment,” ongoing since 1949 [42]. Throughout this long-term experiment, each transect of soil has received 1 of 3 P fertilization treatments: 0 (low), 15 (medium) and 45 (high) Kg[P].Ha^−1^.Year^−1^, resulting in a 3- to 5-fold difference in bioavailable P between the low and high treatments [43]. To differentiate the long-term adaptive effect of P limitation on the microbial community from the effect of short-term changes in P availability, we also fertilized a subset of the low P soil at the time of planting and designated this condition low+P.

**Fig 1:**
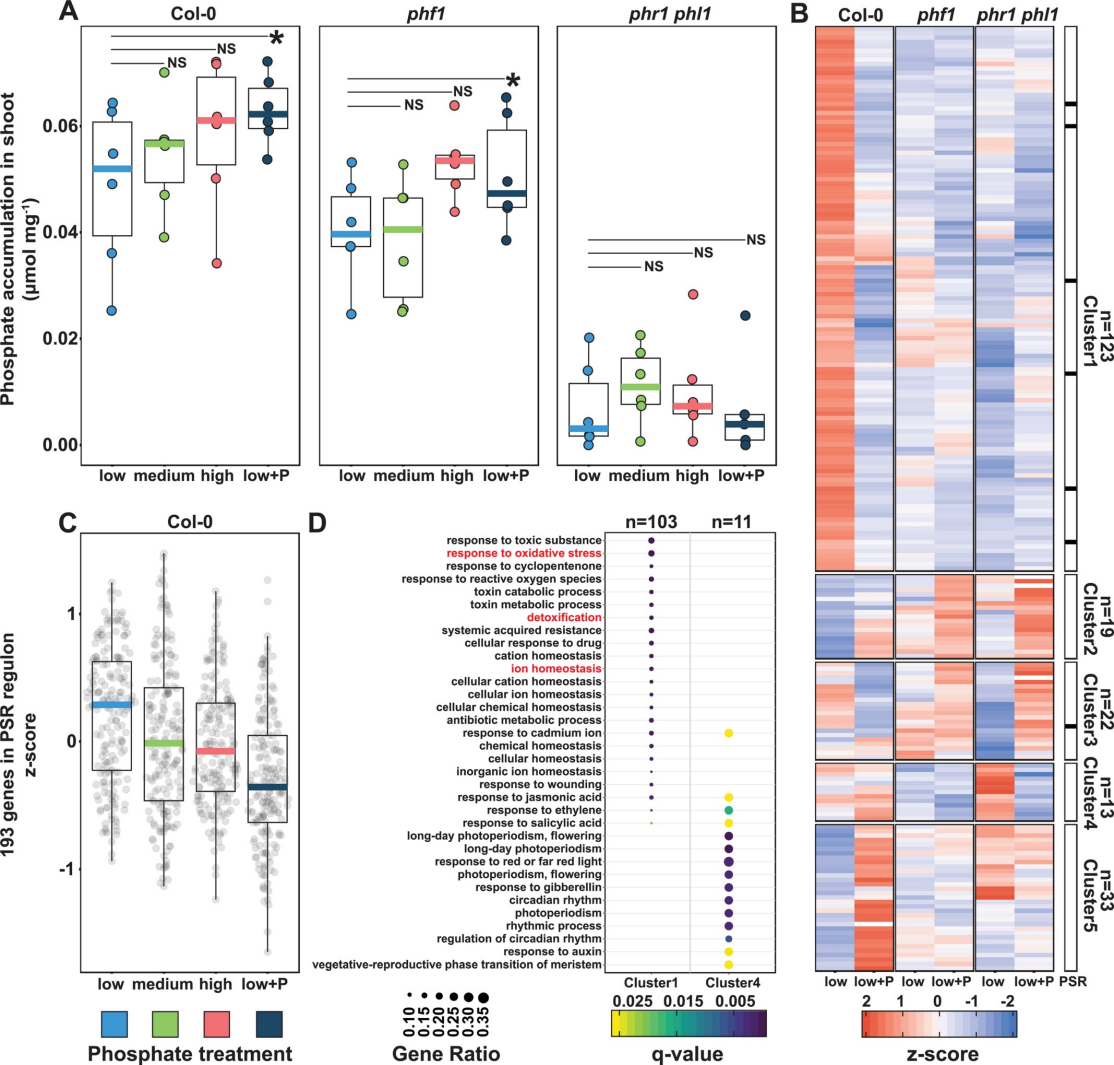
Plants respond to differential P conditions in soil. (A) Free phosphate content normalized by shoot fresh weight (mmol·mg^−1^) across wt Col-0 plants and 2 PSR mutants, *phf1* and *phr1 phl1*. Statistical significance between low P and low+P treatments was determined across each genotype independently by a paired *t* test (*p* < 0.05). (B) Heat map showing the average standardized expression of 210 DEGs across the low P and low+P samples in the Col-0, *phf1* and *phr1 phl1* genotypes. The black bar to the right highlights the distribution of 7 genes belonging to the in vitro defined PSR marker genes [4] across the 5 clusters in the heat map. (C) Average expression of 193 PSR marker genes [4] across the 4 phosphorus regimes in the Col-0 genotype. (D) GO enrichment for Clusters 1 and 4. Clusters 2, 3, and 6 did not show any statistically significant GO enrichment. The gene ratio is the proportion of genes per cluster that belong to a GO category. DEG, differentially expressed gene; GO, gene ontology; P, phosphorus; PSR, phosphate starvation response; wt, wild type.

We examined whether PSR, defined and typically studied in axenic conditions, is active in our soil-based experimental system. We harvested 8-week-old plants grown in the different soils and quantified developmental and molecular phenotypes typically associated with PSR in both wt plants and mutants. We found a strong positive correlation among all developmental features analyzed: shoot area, shoot fresh weight, and shoot Pi accumulation across all soil conditions (Figs 1A and S1A–S1C and S1 Table and S1 Data). In wt plants, shoot Pi accumulation reflected soil P conditions (Fig 1A and S1 Data). As expected [44], *phr1 phl1* showed a dramatic reduction in all phenotypic parameters (Figs 1A and S1B and S1C and S1 Data) and *phf1* accumulated less shoot Pi than wt but did not display any obvious morphological effect (Figs 1A and S1B–S1D and S1 Data).

To identify the transcriptomic signature of PSR in a low P soil, we compared the root transcriptomes of the 3 genotypes from the low P samples with those of the low+P samples (S2 Table). Using a likelihood ratio test, we identified 210 genes that were differentially expressed across genotypes and P conditions (*q* < 0.1). After hierarchical clustering, 123 (59%) of these genes fall into a single cluster (Cluster 1) of co-expressed genes that are exclusively highly expressed in wt under low P and not in either of the PSR mutants (Fig 1B and S1 Data). Thus, these genes represent a PSR under our experimental conditions. A gene ontology (GO) enrichment analysis (Fig 1D and S1 Data) illustrates that these genes are involved in processes such as ion homeostasis, detoxification, and response to oxidative stress. Interestingly, few PSR genes defined from in vitro experiments were significantly differentially expressed in our soil experiment. From a previously defined set of 193 PSR marker genes defined using 7-day-old seedlings exposed in vitro to P limitation for up to 2 days [4], only 7 were called as significant in our experiment using 8-week-old plants. Nevertheless, all 7 of these genes were enriched in wt in low P soil (Fig 1B and S2 Table). Surprisingly, despite the fact that *phf1* and *phr1 phl1* have contrasting transcriptional responses to Pi limitation in vitro [41], the 123 genes in Cluster 1 were not up-regulated in both mutants. To corroborate that the canonical in vitro defined PSR is also induced in wt plants, we compared the median expression of the set of 193 PSR marker genes [4] across the different soils (Figs 1C and S1E and S1 Data). As expected, shoot Pi content was significantly correlated with the induction of PSR marker genes (Figs 1C and S1F and S1 Data), with the highest median expression level in the low P conditions. We conclude that although the response of 8-week-old plants to low P conditions in natural soil is markedly different from in vitro defined PSR, wt plants indeed respond to low P conditions in the soils tested in a Pi concentration- and PHR1/PHL1-dependent manner.

### Bacterial and fungal plant microbiota differ in plant recruitment patterns

**Fig 2:**
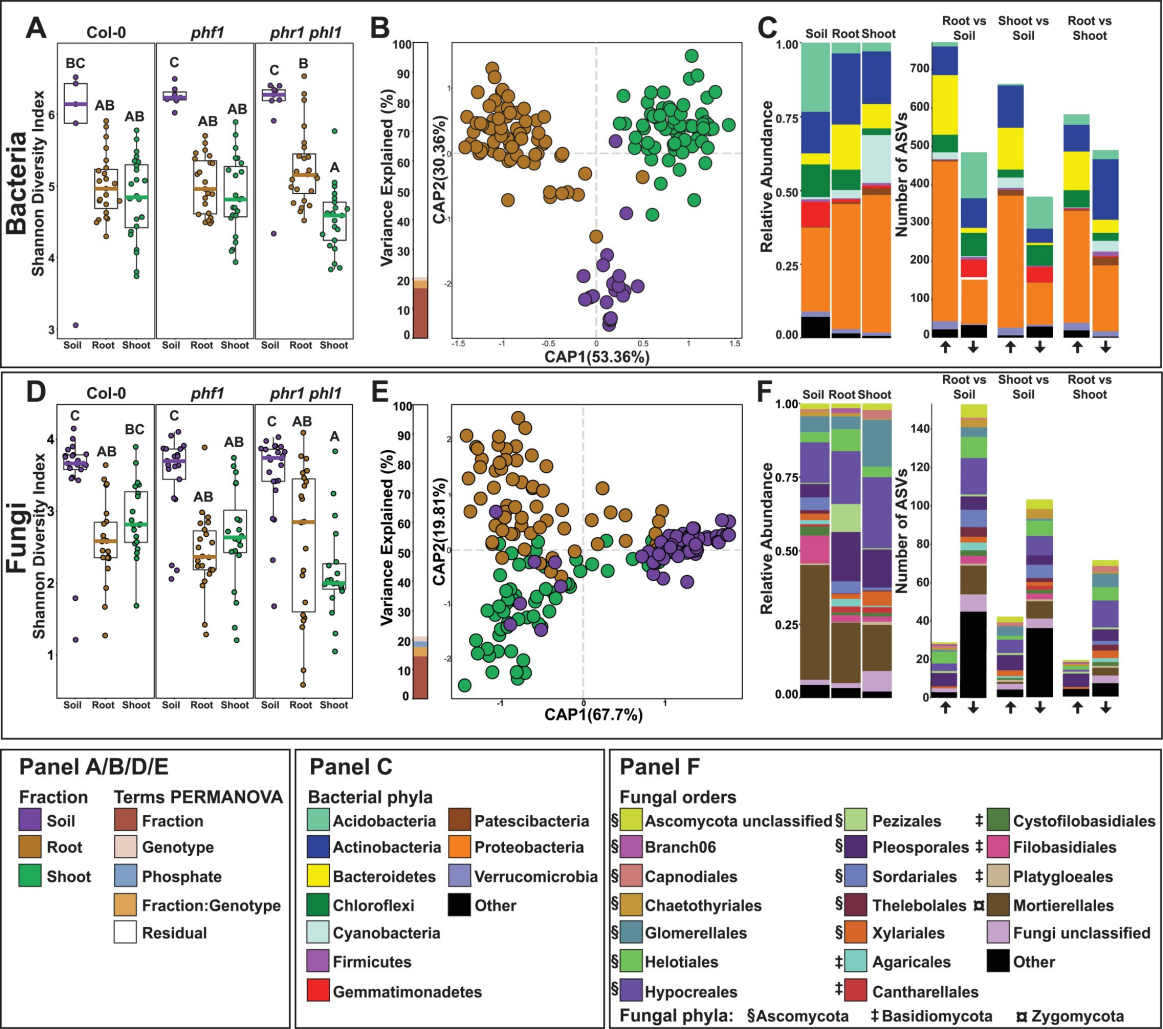
Plant recruitment patterns of bacteria and fungi. (A, D) Bacterial (A) and fungal (D) alpha diversity estimated using the Shannon Diversity Index. Letters represent post hoc test results, based on a full factorial ANOVA model. (B, E) CAP based on Bray-Curtis dissimilarities between bacterial (B) and fungal (E) communities across the soil, root, and shoot. The bar graph to the left of the CAP depicts the percentage of variance explained by statistically significant (*p* < 0.05) terms in a PERMANOVA model. (C) Left panel: Relative abundance profiles of the main bacterial phyla across the soil, root, and shoot fractions. Right panel: Number of statistically significant ASVs enriched in specific fractions. The arrows on the bottom of the panel denote the direction of the enrichment relative to the name of the contrast tested; the up arrow means enrichment in the left fraction of the contrast, whereas the down arrow means enrichment in the right fraction of the contrast (e.g., RootvsSoil, up arrow enriched in root relative to soil, bottom arrow enriched in soil relative to root). A detailed interactive visualization of the bacterial enrichment patterns across the multiple taxonomic levels can be found at https://itol.embl.de/tree/1522316254174701551987253. (F) Left panel: Relative abundance profiles of the main fungal orders across soil, root, and shoot fractions. Right Panel: Number of statistically significant ASVs enriched in specific fractions. The arrows on the bottom of the panel denote the direction of the enrichment relative to the name of the contrast tested; the up arrow signifies enrichment in the left fraction of the contrast, whereas the down arrow signifies enrichment in the right fraction of the contrast (e.g., RootvsSoil, up arrow enriched in root relative to soil, bottom arrow enriched in soil relative to root). Plot is colored by order. The symbols besides the colors in the legend denote phylum. A detailed interactive visualization of the fungal enrichment patterns across the multiple taxonomic levels can be found at https://itol.embl.de/tree/13656172137464831571097084. ASV, amplicon sequence variant; CAP, canonical analysis of principal coordinates; PERMANOVA, Permutational Multivariate Analysis of Variance.

We studied the relationship between PSR and the plant microbiome in wt plants and the 2 PSR mutants grown in all 4 soils. Total DNA was extracted from shoots, roots, and soil, and the 16S rRNA (V3-V4) and ITS1 regions were amplified and sequenced to obtain bacterial and fungal community profiles, respectively. Sequences were collapsed into amplicon sequence variants (ASVs. Bacterial and fungal alpha- and beta-diversity measures conform to previously published data [19,36]: Microbial diversity decreased from the soil to the root and shoot compartments (Figs 2A and 2D and S2A and S2B and S3 Table and S1 Data), and roots and shoots harbor bacterial and fungal communities distinct from the surrounding soil community and from each other (Figs 2B and 2E and S2C–S2F and S4 Table and S1 Data). Plant-derived samples were primarily enriched in comparison to soil with members of the phyla Proteobacteria, Bacteroidetes, and Actinobacteria and depleted in members of Acidobacteria and Gemmatimonadetes (Figs 2C and S2E and S4 Table and S1 Data). Plant-enriched fungal ASVs belonged mainly to the phyla Ascomycota (orders Hypocreales and Pleosporales) and Basidiomycota (order Agaricales). Plant-depleted fungal ASVs belonged mainly to Filobasidiales (Basidiomycota) and Mortierellales (Zygomycota; Figs 2F and S2F and S5 Table and S1 Data).

To quantify the effect of soil community composition on the composition of root and shoot microbiota, we used Mantel tests to detect correlation between dissimilarity matrices of the 3 fractions (root, shoot, and soil). For bacteria, both root and shoot community dissimilarities were strongly correlated with soil community dissimilarity (S3A and S3B Fig and S1 Data), whereas for fungi, no correlation was measured between root and soil (S3D Fig and S1 Data), and only a weak correlation was measured between shoot and soil (S3E Fig and S1 Data). This observation indicates that both root and shoot bacterial communities are strongly dependent on soil community composition, despite the fact that bacterial microbiota are distinct from the soil community (Fig 2B and S1 Data). By contrast, the fungal microbiota composition both above and below ground is independent of relative abundances within the soil inoculum. This difference implies that the plant’s microbiota filtering mechanisms are fundamentally different for fungi and bacteria.

### Shoot and root microbiota are both correlated and distinct

Shoot and root microbiomes are linked, and substantial crosstalk is expected to occur between these 2 niches [45,46]. We show here that roots and shoots harbor distinct communities from each other (Figs 2B and 2E and S2C–S2F and S4 Table and S1 Data). To further explore organ specificity in the plant microbiome composition, we compared root and shoot samples at the ASV level. Shoots were mainly enriched with the bacterial phyla Cyanobacteria and Patescibacteria compared to the root, whereas roots were enriched with Proteobacteria, Chloroflexi, and Bacteroidetes (Figs 2C and S2E and S4 Table and S1 Data). With regard to fungal orders, shoots were enriched with Capnodiales, Glomerellales, Pleosporales, and Hypocreales, whereas roots were enriched with Pezizales, Helotiales, and Mucorales (Figs 2F and S2F and S5 Table and S1 Data). The shoot enrichment of Cyanobacteria suggests that the availability of light is an important factor in niche differentiation within the plant [47–49]. We used Mantel tests to detect correlation between dissimilarity matrices of root and shoot samples. Despite the fact that they harbor distinct communities, roots and shoots were correlated with each other for both bacteria and fungi (S3C and S3F Fig and S1 Data). Thus, although roots and shoots form distinct bacterial and fungal niches, shifts in microbiota in both of these niches are correlated, suggesting that either one or both of these fractions serves as an inoculum source for the other.

### The plant microbiome composition is driven by the plant PSR status

**Fig 3:**
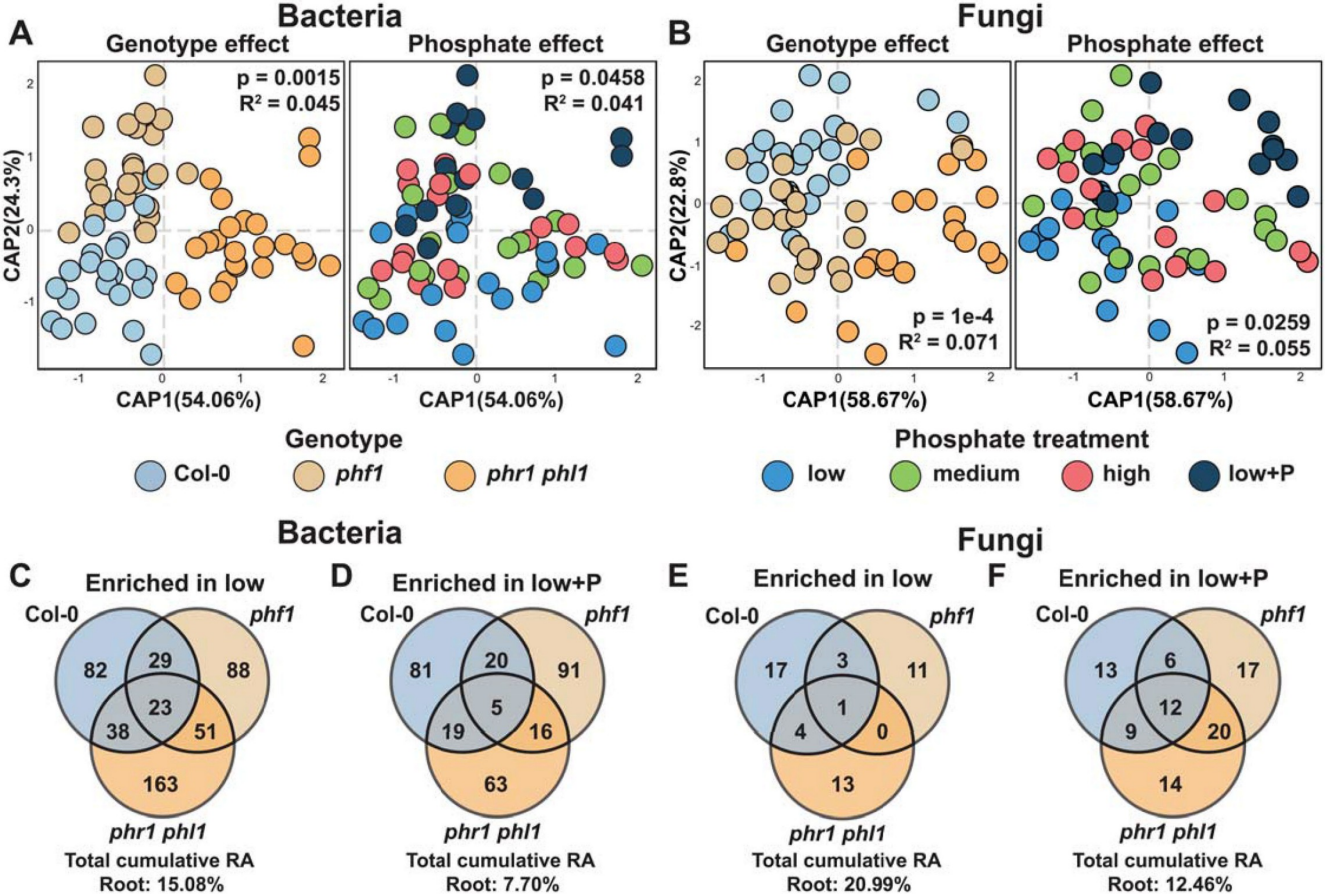
Plant PSR controls the assembly of the plant microbiome. (A, B) Canonical analysis of principal coordinates showing the influence of plant genotypes and soil P content over the (A) bacterial and (B) fungal communities in the root. The *p*-value and R^2^ values inside each plot are derived from a PERMANOVA model and correspond to the genotype and P term, respectively. (C, E) Venn diagrams showing the distribution of (C) bacterial and (E) fungal ASVs with statistically significant (*q* < 0.1) higher abundance in the low P treatment in comparison to the low+P treatment in the Col-0, *phf1* and *phr1 phl1* roots. (D, F) Venn diagrams showing the distribution of (D) bacterial and (F) fungal ASVs with statistically significant (*q* < 0.1) higher abundance in the low+P treatment in comparison to the low P treatment across the Col-0, *phf1* and *phr1 phl1* roots. ASV, amplicon sequence variant; P, phosphorus; PERMANOVA, Permutational Multivariate Analysis of Variance; PSR, phosphate starvation response; RA, relative abundance.

We investigated the influences of plant PSR signaling and the different soil P concentrations on microbial community composition. Constrained ordination showed significant differences, explaining a similar proportion of variance, in both bacterial and fungal community compositions. These differences persist across the P accumulation gradients represented by both the difference in soil P and PSR mutants (Figs 3A and 3B and S4A and S4B and S1 Data). This effect is maintained also when considering 13 soil edaphic factors that were measured in for the same soil samples [43] (S5A–S5D Fig and S1 Data). For both bacteria and fungi, the PSR genotype effect in roots was more consistent than the soil P effect (Fig 3A and 3B and S1 Data), which was mainly driven by the P-amended low+P samples. In shoots, both bacteria and fungi responded to PSR genotype but in this case did not respond to soil P (S4C and S4D Fig and S1 Data). We did not observe a significant soil P:genotype interaction effect for either bacteria or fungi (S4A and S4B Fig and S1 Data), confirming that *phf1* and *phr1 phl1* both had atypical bacterial microbiomes regardless of Pi status. As expected, we did not observe a PSR effect in the soil samples (S4E and S4F Fig and S1 Data). The notable genotype effect illustrates that the plant niche filtering (Fig 2B and 2E and S1 Data) is partly shaped by PSR.

To define which taxa at the ASV-levels were influenced by soil P and/or plant PSR, we applied a generalized linear model (GLM) to the count datasets (S6 and S7 Tables). Contrasting the low P samples against the low+P samples, we detected 769 bacterial (S6 Table) and 140 fungal (S7 Table) ASVs, accounting for 23% and 33% of the bacterial and fungal abundance in the root, respectively, that were differentially abundant in at least 1 genotype (Fig 3C–3F and S1 Data). Of these, most (568 bacterial and 85 fungal ASVs) were genotype specific, suggesting that the Pi response of these taxa is not direct but is rather driven by Pi responses in the plant. Taken together, these results indicate that plant microbiota are relatively robust to differences in soil P content but are sensitive to the plant PSR status. Responses to soil P concentration are contingent on PSR regulatory elements under both low and high P conditions.

### Bacterial synthetic communities modulate the plant PSR

**Fig 4:**
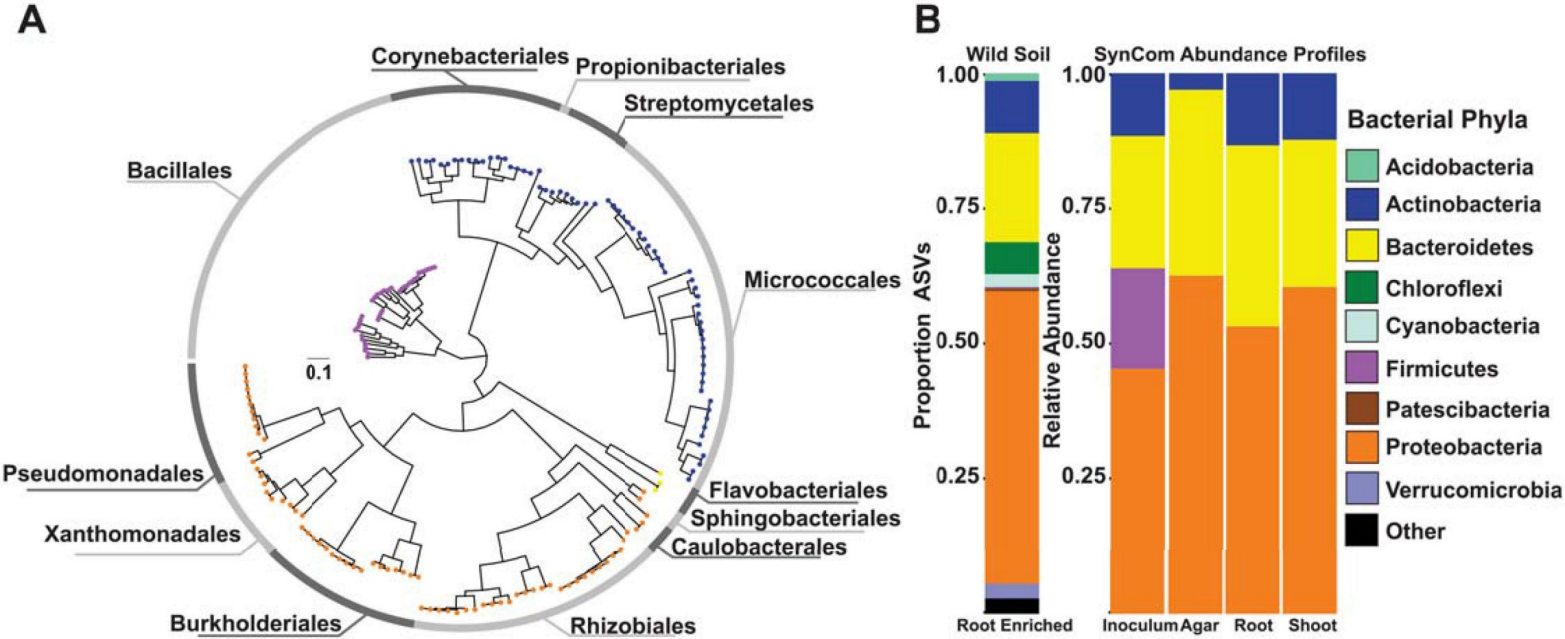
Bacterial SynCom reproduces the typical plant-associated taxonomic distribution found in soil. (A) Phylogenetic tree of 185 bacterial genomes included in the SynCom. The tree tips are colored according to the phylum classification of the genome in panel B; the outer ring shows the distribution of the 12 distinct bacterial orders present in the SynCom. (B) Left Panel: Proportion of ASVs enriched in the root in comparison to the natural soil across all treatments and genotypes based on a fitted GLM (*q* < 0.1). Each ASV is colored according to its phylum-level classification. Right Panel: Relative abundance profiles of bacterial isolates across the initial bacterial inoculum, planted agar, root, and shoot fractions. Each isolate is colored according to its phylum-level classification based on the genome-derived taxonomy. ASV, amplicon sequence variant; GLM, generalized linear model; SynCom, synthetic community.

The results obtained from the soil experiment suggest that the community structure of the plant microbiome is not only determined by first-order interactions (plant–microbe, microbe–microbe, microbe–environment) but also by higher-order interactions, such as the effect of abiotic conditions on plant–microbe interactions. This is evident in the large proportion of ASVs that respond to soil P in a genotype-specific manner (Fig 3C–3F and S1 Data). To establish a system in which interactions of different orders of complexity can be studied reproducibly, we constructed a plant–microbe microcosm that can be deconstructed to its individual components while retaining a complexity that is comparable to natural ecological communities. We designed a representative bacterial SynCom from a culture collection composed of isolates derived from surface-sterilized *Arabidopsis* roots [50]. We selected 185 genome-sequenced isolates representing a typical plant-associated taxonomic distribution (Fig 4A and 4B and S1 Data). We grew each isolate separately and mixed the grown cultures to equal optical densities. We grew 7-day-old *Arabidopsis* seedlings in a Pi concentration gradient (0, 10, 30, 50, 100, 1000 μM KH_2_PO_4_) and concomitantly exposed them to the SynCom on vertical agar plates for 12 days.

**Fig 5:**
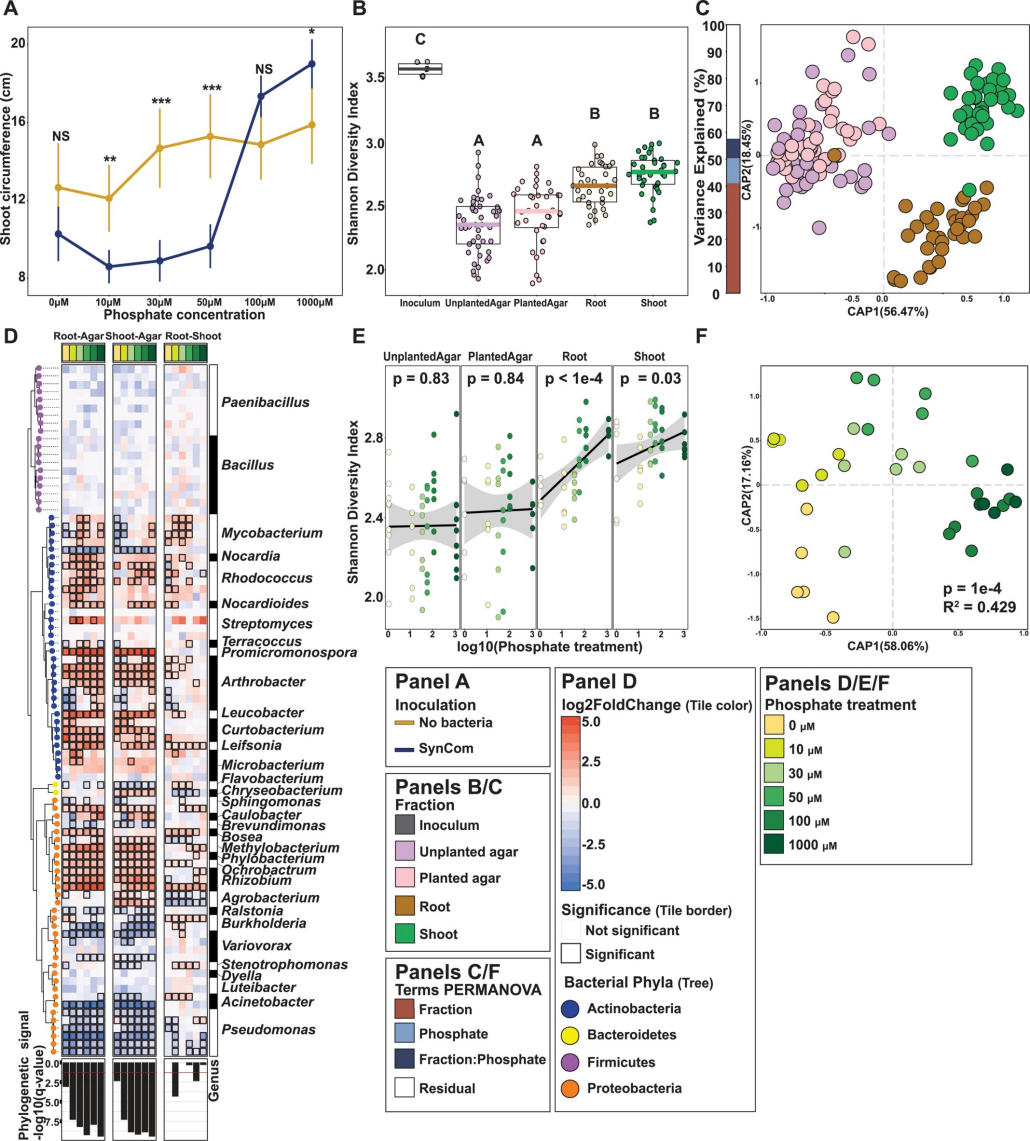
Synthetic bacterial communities display deterministic community assembly in plants. (A) Strip chart displaying the average shoot size of Col-0 *Arabidopsis* grown across a Pi gradient either in sterile conditions or with the SynCom. Each dot in the scatter plot represents the mean value for that particular treatment; the range crossing each dot represents the 95% confidence interval calculated. The lines are drawn to connect the means. (B) Alpha diversity across the fractions sampled was estimated using the Shannon Diversity index. An ANOVA model followed up by a Tukey HSD test were applied to estimate differences between inoculum, unplanted agar, planted agar, root, and shoot fractions. Letters represent the results of the post hoc test. (C) CAP based on Bray-Curtis dissimilarities between bacterial communities across the 4 fractions sampled. The bar graph to the left of the CAP depicts the percentage of variability explained by statistically significant (*p* < 0.05) terms in the PERMANOVA model. (D) Enrichment patterns of the SynCom. Each row along the different panels of the figure represents a USeq: a USeq encompasses a set of indistinguishable V3-V4 16S rRNA sequences present in the 185-member SynCom. Phylogenetic tree (on the left) is colored based on the phylum-level classification of the corresponding USeq. Each column in the heat maps represents a specific contrast in the enrichment model. We calculated root versus agar (left heat map), shoot versus agar (middle heat map), and root versus shoot (right heat map) enrichments within each Pi treatment (e.g., Root_0Pi versus Agar_0Pi). The heat maps are colored based on log_2_ fold changes derived from the fitted GLM. Positive fold changes (colored in red gradient) represent enrichments on the left side of the name of the contrast (e.g., Root-Agar, enriched in root in comparison to agar), whereas negative fold changes (colored in blue gradient) represent enrichments on the right side of the name of the contrast (e.g., Root-Agar, enriched in agar in comparison to agar). Boxed cells represent statistically significant enrichment/depletion. The bottom panel depicts the transformed (−log_10_) *q*-value derived from a phylogenetic signal Pagel’s λ test. Tests were performed per column in the heat map (e.g., Root0μM Pi versus Agar0μM Pi). (E) Bacterial alpha diversity estimated using the Shannon Diversity index. *p*-values derived from a linear model are shown for each fraction. Linear regression line is shown in black and the 95% confidence interval is shaded in gray. (F) CAP showing the influence of phosphate on the bacterial communities in the root. The bar graphs to the left of the CAP depict the percentage of variability explained by statistically significant (*p* < 0.05) variables based on a PERMANOVA model. CAP, canonical analysis of principal coordinates; GLM, generalized linear model; HSD, Honestly Significant difference; PERMANOVA, Permutational Multivariate Analysis of Variance; Pi, orthophosphate; SynCom, synthetic community; USeq, unique sequence.

First, we investigated whether PSR is induced in our experimental system. Similar to the natural soil-based experiment, we quantified developmental and transcriptional phenotypes associated with PSR in plants grown in different concentrations of Pi. In line with the work by Castrillo and colleagues [4], the presence of the SynCom consistently decreased primary root elongation across all Pi concentrations compared with the uninoculated control, but the Pi gradient did not affect this parameter (S6A Fig and S1 Data). Shoot size increased with Pi concentration, and the slope of this trend was affected by the presence of the SynCom: At high Pi, the SynCom tended to increase shoot size, whereas at low Pi the SynCom decreased it (Fig 5A and S1 Data), suggesting that the microbiome plays a role in shaping the plant’s response to different Pi concentrations.

We performed RNA sequencing (RNA-Seq) on inoculated and uninoculated seedlings exposed to high (1,000 μM) and low (50 μM) Pi. To confirm that our low Pi treatments induce PSR, we examined the expression of the 193 PSR markers defined in the work by Castrillo and colleagues [4]. We found that 168 of the 193 PSR markers genes were significantly induced in uninoculated plants at low Pi compared with high Pi conditions. In the presence of the SynCom, 184 out of 193 PSR marker genes were significantly induced, and the average fold change increased from 4.7 in uninoculated conditions to 11 in the presence of the SynCom (S6B Fig and S1 Data). We further examined whether the 123 low Pi-responsive genes from the soil experiment (Cluster 1 in Fig 1B) are overexpressed in the agar system as well. We found that 59 of the 123 genes (47.2%) were low Pi-enriched in uninoculated plants and 72 (58.5%) were low Pi-enriched in the presence of the SynCom. The average fold change for this set of 123 genes was 1.6 in uninoculated conditions and 2.0 in the presence of the SynCom (S6C Fig). These results confirm that (i) in both our systems, wild soils and axenic conditions, PSR is induced at low Pi, and (ii) the SynCom enhances this induction, similar to the results reported in the work by Castrillo and colleagues [4].

### Bacterial SynComs display deterministic community assembly in plants

To quantify the establishment of the SynCom in the plants, we determined bacterial community composition after 12 days of co-inoculation in roots, shoots, and agar via 16S rRNA gene amplicon sequencing, mapping reads to 97 unique sequences (USeqs) representing the 185-strain SynCom (S8 Table). We found that plant roots and shoots sustained a higher bacterial alpha diversity than the surrounding agar (Fig 5B and S9 Table and S1 Data), an aspect in which our experimental system differs from a natural environment where species richness is higher in the surrounding soil than in the plant (Fig 2A and S1 Data). As in natural soil experimental systems, agar, roots, and shoots assembled distinct bacterial communities, and this difference among these 3 fractions explained most of the variance in community composition despite the different Pi concentrations (Figs 5C and S6D and S1 Data).

To study which strains are enriched in the roots and shoots under the different Pi concentrations, we utilized a GLM (S10 Table). Noticeably, plant (root and shoot) enrichment is strongly linked to phylogeny (Fig 5D and S1 Data) and is robust across the phosphate gradient assayed. In contrast, the root versus shoot comparison did not exhibit a significant phylogenetic signal, highlighting the fact that the ability to differentially colonize the shoot from the root under these conditions is phylogenetically scattered across the SynCom. As in the soil census, shoot, root, and agar beta diversities were significantly correlated (S6E–S6G Fig and S1 Data).

We hypothesized that by establishing a standardized protocol for producing the inoculum and controlling the growth conditions, we will have created a reproducible system in which most of the variance can be accounted for. To test this, we compared the number of ASVs and total relative abundance captured by the fraction (root/shoot versus soil/agar) in a GLM in the natural community experiment versus the SynCom experiment. Supporting our hypothesis, only 1,518 out of 3,874 measurable ASVs (32% of the total ASVs), accounting for 72% of the relative abundance in plant tissue, shift significantly between root and soil in the natural community survey, whereas 58 out of 97 USeqs (59%), accounting for 99% relative abundance in plant tissue, were significantly enriched or depleted in plant tissue in the SynCom experiment (Fig 5D and S1 Data).

These results indicate that plant colonization is largely deterministic in our SynCom system, in comparison to microbiomes in nature. The reproducibility of this system, coupled with our ability to edit it as a tool for hypothesis-testing, is crucial to bridge ecological observation with mechanistic understanding of plant–microbiota interactions.

### P stress-induced changes in the root microbiome

The shifting role of the SynCom from increasing shoot size under replete Pi to decreasing shoot size and PSR induction under Pi limitation (Figs 5A and S6B and S1 Data) can be explained by either a shift in the lifestyle of individual bacteria along the mutualist–pathogen continuum or by changes in the microbiota composition along the Pi gradient. The latter would favor the proliferation of mutualist bacteria only when sufficient nutritional requirements are met. To measure the effect of the Pi concentration in the media on the SynCom composition in wt plants, we measured alpha and beta diversity along our Pi gradient (0, 10, 30, 50, 100, 1000 μM KH_2_PO_4_) in roots, shoots, and agar. We observed a positive correlation between alpha diversity and Pi concentrations, resembling a partial ecological diversity–productivity relationship—the prediction/observation of a bell-shaped response of ecological diversity to environmental productivity [51,52]—in roots and shoots but not in the surrounding agar (Fig 5E and S1 Data). As for beta diversity, the composition of the SynCom shifted significantly along the Pi concentration gradient (Figs 5F and S7A–S7E and S1 Data). Pi concentration therefore alters the plant microbiome, shifting from a net-positive outcome for the plant to a net-negative one as measured by shoot size (Fig 5A and S1 Data).

### *Burkholderia* respond to Pi stress-induced changes in the plant

**Fig 6:**
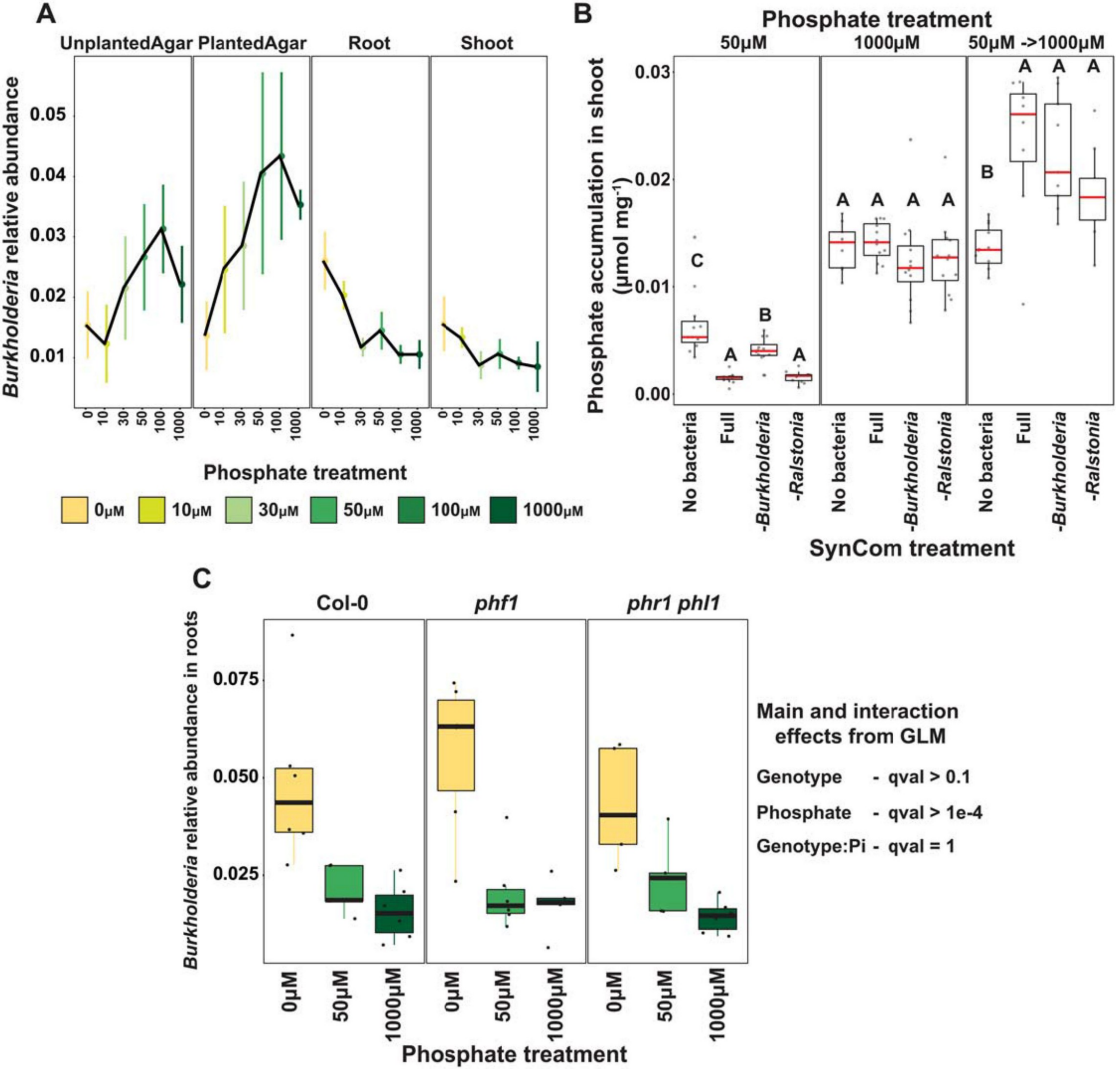
Bacterial strains respond to Pi-stress-induced physiological changes in the wt plants. (A) Relative abundance of *Burkholderia* Useqs, both of which exhibit a statistically significant (*q* < 0.1) Pi-enrichment between the plant fractions and the agar fraction. The middle dot of each strip bar corresponds to the mean of that particular condition, the range of the strip bar corresponds to the 95% confidence interval of the mean. The lines are drawn connecting the means for each Pi concentration. (B) Box plots showing the phosphate accumulation in plants exposed to different SynComs across 3 phosphate treatments. Statistically significant differences among SynCom treatments were computed within each phosphate treatment separately using an ANOVA model. Letters represent the results of the post hoc test. (C) Box plot showing relative abundance of *Burkholderia* USeqs across 3 Pi concentrations and 3 plant genotypes. Summary of the NB-GLM for *Burkholderia* is shown on the right. GLM, generalized linear model; NB, no bacteria; Pi, orthophosphate; SynCom, synthetic community; USeq, unique sequence; wt, wild type.

In a previous publication [4], we demonstrated that PHR1 negatively regulates defense-related genes under low Pi conditions. Suppression of plant defense and consequent alterations in colonization could account for some of the shift we observed from a beneficial to a detrimental community. We thus aimed to identify bacteria that respond to Pi stress-induced changes in the plant, rather than the Pi concentration itself. To do so, we searched for USeqs that displayed a strong Pi:fraction (shoot, root, agar) interaction in our GLM (S8A Fig and S11 Table and S1 Data). Two of the three USeqs displaying the strongest Pi:fraction interaction belonged to *Burkholderiaceae*, representing all 5 *Burkholderia* strains used in this experiment. The relative abundance of these USeqs is positively correlated with Pi concentration in the agar but is negatively correlated with Pi concentration in the root and shoot (Figs 6A and S8B and S8C and S1 Data). This pattern suggests that these strains are responding to physiological changes in the plant.

To measure the physiological effect of the specific recruitment of *Burkholderia* under Pi stress on the plant, we conducted a drop-out experiment in which we compared plants inoculated with the full SynCom to plants inoculated with a SynCom excluding all 5 *Burkholderia* isolates. We also included a SynCom excluding all members of the neighboring *Ralstonia* clade (Fig 4A and S1 Data), which did not display any discernible Pi response. We measured shoot size and Pi concentrations in the shoots (a proxy for PSR) of plants grown in high (1,000 μM) and low (50 μM) Pi with the different SynComs. In addition, we measured shoot size and Pi content in a refeeding treatment with SynCom-inoculated plants grown in low (50 μM) Pi and then transferred to high Pi (1,000 μM) conditions. As seen before (Fig 5A and S1 Data), the SynCom decreased shoot sizes in low Pi and increased them in high Pi. However, the different taxon drop-outs did not affect shoot size compared with the full SynCom, except for a slight decrease in post-refeeding shoot size in the *Ralstonia* drop-out treatment (S9 Fig and S1 Data). All SynCom treatments decreased shoot Pi content in the low Pi conditions compared with the uninoculated plants but recovered to a higher shoot Pi level than the uninoculated treatments upon transferring to high Pi conditions, reproducing our previous report [4] (Fig 6B and S1 Data). Among inoculated treatments, plants colonized with the *Burkholderia* drop-out treatment (SynCom excluding all *Burkholderia*) had a higher Pi content than either plants colonized with the full SynCom or with the *Ralstonia* drop-out SynCom only in the low Pi conditions. There was no difference in shoot Pi among the SynCom treatments in either the high Pi treatment or following the refeeding treatment. This finding suggests that the enrichment of *Burkholderia* in plant tissue under Pi starvation can be considered a shift in the effect of bacteria on the plant from a positive interaction to a negative one.

To test whether *Burkholderia* are recruited to the plant under low Pi via a PSR-dependent mechanism, we inoculated 7-day-old wt, *phf1*, and *phr1 phl1* seedlings with the SynCom and profiled the community composition after 12 days of growth in 3 Pi concentrations (0, 50, and 1,000 μM). In accordance with our soil experiment, and with the work by Castrillo and colleagues [4], community composition assembled in the roots of the 3 genotypes differed significantly (S10 Fig and S1 Data). However, *Burkholderia* sequences were enriched in low Pi in all 3 genotypes (Fig 6C and S1 Data), indicating that their recruitment to the root under low Pi is independent of PSR activation and of the immune dampening that accompanies it.

## Discussion

This study shows that despite 60 years of differential fertilization, the plant’s PSR and accompanying changes to its microbiome composition between the low P and high P soils are subtle, possibly because Pi status of the plant is highly buffered by the plant ionomic regulatory network [53]. Only when comparing the low P versus the P-supplemented low+P samples is there a discernible difference in both plant transcriptome and shoot Pi accumulation, which correlates to a stronger effect on microbiota composition. This suggests that bioavailable Pi added to the soil is quickly consumed, and short-term amendments are needed in order to detect changes. A similar short-term fertilization approach was successfully applied in the work by Fabiańska and colleagues [36], using soils from a different long-term experimental site.

As opposed to in vitro defined transcriptomes [41], here, *phf1* and *phr1 phl1* plants, although each having a distinct transcriptional profile, were impaired in the response of a core set of genes (Cluster 1 in Fig 1B) to low P. This is despite the fact that *phf1* plants were not impaired in growth (S1B and S1C Fig and S1 Data) and were only slightly impaired in shoot Pi accumulation (S1D Fig and S1 Data). A paralogous discrepancy was also observed in the work by Hiruma and colleagues [17], in which both *phf1* and *phr1 phl1* were impaired in their response to a plant growth promoting fungus, whereas only *phr1 phl1* but not *phf1* was impaired in P translocation to the shoot, raising the speculation that PHF1 is needed for fine, rather than bulk, P translocation within the plant. Our results support this idea and raise a similar speculation that PHF1-guided transporters are involved in fine grained phosphate translocation within the plant toward Pi sensing tissues.

An additional apparent departure from the axenically defined PSR transcriptional signature is that only 7 of the genes that were induced in wt plants under low Pi conditions overlapped with the list of 193 axenically defined PSR genes we used as a reference. It is notable, however, that despite the difference in plant age and environmental conditions between our soil and axenic agar systems, the agar-defined transcriptomic signature is detectable in the soil system (Fig 1C and S1 Data) and vice versa: the signature of the 123 genes identified in our soil system is detectable on agar (S6C Fig and S1 Data). Thus, despite the apparent differences, agar-defined PSR signatures are also maintained under complex real-life conditions.

Several studies link host physiological response to the soil phosphate status with the bacterial [4,38] and fungal [34,36] microbiome. A recent report of *Arabidopsis* planted in a 60-year-long annual P fertilization gradient (the same soil used in the current study) showed a modest P effect on plant microbiome composition [43]. Previously, we showed that PSR mutants in *Arabidopsis* have different bacterial microbiomes in Pi replete conditions [4], and a recent publication showed that PSR mutants had a slightly altered fungal microbiome in Pi-replete but not in Pi-depleted conditions [36]. Here, we analyzed fungi and bacteria side by side and demonstrated a pronounced effect of PSR impairment on both bacterial and fungal components of the plant microbiota. We noted an intriguing difference that emerged in the patterns of community assembly between bacteria and fungi (S3A, S3B, S3D and S3E Fig and S1 Data). The bacterial microbiota composition is strongly dependent on the soil bacterial community composition, whereas changes to the fungal microbiota are uncoupled from changes to the soil fungal community composition. This indicates that the plant is markedly more selective as to the fungi allowed to proliferate in its tissue than it is with bacteria. The observation that much of the modulation of the plant’s fungal microbiota is mediated by the bacterial microbiota itself [54] may also contribute to this complex pattern. Our results show that impairment of PSR genes profoundly affects the composition of the plant microbiota, independently of P conditions, and that observed shifts in root-derived microbial communities may not be a result of sensitivity to P concentrations but rather a response to PSR regulation in the hosts. The mechanism by which PSR regulation affects microbiota assembly is not fully understood. On one hand, PSR and plant immunity have been shown to be transcriptionally linked [4]. On the other, Pi depletion drastically changes the root’s exudate profiles [17,38,39,55], which have been shown to play a critical role in plant microbiota assembly [56,57]. It is likely that both factors contribute to shifts in microbiota composition. Evidently, however, the *Burkholderia* plant-enrichment in our SynCom occurs via a PSR-independent mechanism.

Our SynCom, comprising 185 genome-sequenced endophytic bacterial isolates, was designed to resemble a natural bacterial community (Fig 4B and S1 Data). The community assembly patterns shown for this system are highly reproducible, demonstrating that microbiome assembly is largely a deterministic process. The reproducibility and editability of this system is attractive for detailed mechanistic study of the processes that determine community assembly and its influence on plant phenotype and fitness. From within this SynCom, the genus *Burkholderia* emerges as a PSR-independent, low Pi-responsive taxon. We compared the effect of *Burkholderia* on shoot Pi accumulation from within a full SynCom (a realistic proxy for the bacterial community) to that of the full SynCom lacking *Burkholderia*, a strategy akin to knocking-out a gene of interest, also recently applied in the work by Durán and colleagues [54]. The control treatment for this type of approach is the full SynCom, whereas in a plant–bacterium binary association experiment it would typically be sterile conditions. Because both sterile conditions and binary association are strong deviations from conditions that may be encountered in the field, the results of binary association experiments may be correspondingly distorted. Using the drop-out approach, we expect to see more subtle differences, because the microbial load on the plant does not change much, but also that these differences be more relevant to the field—an expectation that is yet to be empirically tested. Our observation that dropping *Burkholederia* out of the SynCom increased shoot Pi in Pi-limiting conditions (50 μM Pi) but not in Pi-replete conditions (1,000 μM Pi) suggests that strains in this genus shift their relationship with the plant from a seeming commensal to a competitor/pathogen. Having ruled out PSR-dependent processes, another plausible explanation for *Burkholderia* enrichment patterns is that when Pi is limiting in the media, the plant becomes a source of Pi for the bacteria and strains with an enhanced ability to utilize plant-derived organic Pi or polyphosphate have an advantage under these conditions. Strains belonging to the genus *Burkholderia* have been shown to be particularly efficient at polyphosphate accumulation at pH 5.5, which is similar to the pH we used in our media [58].

Shifts in microbiota composition that accompany PSR are either adaptive to the plant or reflect opportunistic strategies by bacteria [4,17]. Under the former hypothesis, microbes recruited by the plant under Pi stress provide the plants with an advantage vis-a-vis coping with this stress, whereas under the latter, opportunistic microbes might be making a bad situation worse for the plant. In the case of *Burkholderia* in our SynCom, results support the latter hypothesis. *Burkholderia* contribute to depletion of shoot Pi stores, only under Pi-limiting conditions. However, plant-adaptive microbial recruitment under low Pi has been shown to occur as well [17]. The fact that bacteria responding to PSR genes are not a monophyletic group in soil indicates that multiple mechanisms are involved in community assembly. It is likely that these mechanisms encompass both plant-adaptive and opportunistic strategies.

## Materials and methods

### Soil P gradient experiment

#### Collection of soil from field site

Soil used in this experiment was collected from the long-term Pi fertilization field (“Field D”) trial at the Julius Kühn Experimental Station at Martin Luther University of Halle-Wittenberg (51°29′45.6′′N, 11°59′33.3′′E) [42,59]. Soil cores (10 cm diameter × 15 cm depth) were taken from 18 6 × 5 m unplanted plots belonging to 2 strips. These plots represent 3 P fertilization regimens: low, medium, and high P (0, 15, and 45 kg P ha^−1^ year^−1^, respectively). Differences in soil mineral content between strips and P fertilization regimens are reported in the work by Robbins and colleagues [41], showing that the different Pi regiments significantly differed only in P content. Soil cores were harvested in the middle of March 2016 (strip 1) and beginning of April 2016 (strip 2). Approximately 2 cm of the topsoil was discarded, and the remaining lower 13 cm of soil was stored at 4°C until use. Soils from each core were homogenized separately with a mesh sieve wire (5 × 5 m^2^). The sieved soil cores were stored at 4°C until use. About 300 g of soil were added to each pot (7 × 7 × 7 cm^3^).

#### Experimental design

Each of the 3 *Arabidopsis* genotypes was grown in soil from all 18 plots (6 plots per P treatment). In addition, a fourth P regimen designated “Low+P” was created by adding additional P to a set of pots with low P. The amount of P added to these pots is based on the difference in total P between low and high P plots. The average difference between low and high P over all the plots is 42 mg P per kg soil [43]. Per pot, this is 12.6 mg P (accounting for 300 g soil per pot). Thus, a 10 ml solution consisting of 4.2 mg P in the form of 20% K_2_HPO_4_ (MilliporeSigma, St. Louis, MO) and 80% KH_2_PO_4_ (MilliporeSigma, St. Louis, MO) was added to the pots in 3 applications (Weeks 2, 4, and 6) before watering (in order to distribute the P through the soil).

Thus, the experiment included 2 variables: soil treatment (low P, medium P, high P, low+P) and genotypes (Col-0, *phf1*, and *phr1 phl1*) with 6 independent replicates, amounting to 72 pots. Pot positions in the greenhouse were randomized.

#### Plant growth conditions

*Arabidopsis thaliana* ecotype Col-0 and mutants *phf1 and phr1 phl1* (both in the Col-0 background) were used. Seeds were surface sterilized (20 min 70% EtOH (MilliporeSigma, St. Louis, MO), 10 s 100% EtOH) and planted directly onto moist soil. Sown seeds were stratified for 3 days at 4°C before being placed in a greenhouse under short-day conditions (6/18 day-night cycle; 19 to 21°C) for 8 weeks. Germinating seedlings were thinned to 4 plants per pot.

#### Sample harvest

After 8 weeks of growth, pots were photographed, and shoot size was quantified using WinRhizo software (Regent instruments Inc., Québec, Canada). Samples were harvested in random order to avoid any confounding circadian effect on the results. For DNA extraction, 2 roots, 2 shoots, and soil from each pot were harvested separately. Roots and shoots were rinsed in sterile water to remove soil particles, placed in 2 ml Eppendorf tubes (Eppendorf, Hamburg, Germany) with 3 sterile glass beads (MilliporeSigma, St. Louis, MO), then washed 3 times with sterile distilled water to remove soil particles and weakly associated microbes. Root and shoot tissue were then pulverized using a tissue homogenizer (TissueLyser II; Qiagen, Hilden, Germany) and stored at −80˚C until processing. Five ml of soil from each pot was suspended in 20 ml of sterile distilled water. The resulting slurry was sieved through a 100 μm sterile cell strainer (Fisher Scientific, Hampton, NH) and the flow-through was centrifuged twice at maximum speed for 20 minutes, removing the supernatant both times. The resulting pellet was stored at −80˚C until processing. For RNA extraction, one root system and one shoot were taken from 3 replicates of each treatment, washed lightly to remove soil particles, placed in 2 ml Eppendorf tubes with 3 glass beads and flash frozen with liquid nitrogen. Tubes were stored at −80˚C until processing. For shoot Pi measurement, 2 to 3 leaves from the remaining shoot in each pot were placed in an Eppendorf tube and weighed; 1% acetic acid (MilliporeSigma, St. Louis, MO) was then added, and samples were flash frozen and stored at −80°C until processing. The Ames method [60] was used to determine the phosphate concentration in these samples.

#### DNA extraction

DNA extractions were carried out on ground root and shoot tissue and soil pellets, using the 96-well-format MoBio PowerSoil Kit (MoBio Laboratories; Qiagen, Hilden, Germany) following the manufacturer’s instruction. Sample position in the DNA extraction plates was randomized, and this randomized distribution was maintained throughout library preparation and sequencing.

#### RNA extraction

RNA was purified from plant tissue using the RNeasy Plant Mini Kit (Qiagen, Hilden, Germany) according to the manufacturer’s instructions and stored at −80˚C.

### Bacterial SynCom experiment

#### Bacterial isolation and culture

The 185-member bacterial SynCom contained genome-sequenced isolates obtained from Brassicaceae roots, nearly all *Arabidopsis*, planted in 2 North Carolina, US, soils. Because both bacteria and fungi responded similarly to PSR in our soil experiments, we only included bacteria, which are more compatible with our experimental system in our SynCom. A detailed description of this collection and isolation procedures can be found in the work by Levy and colleagues [50]. One week prior to each experiment, bacteria were inoculated from glycerol (MilliporeSigma, St. Louis, MO) stocks into 600 μL KB medium in a 96 deep well plate. Bacterial cultures were grown at 28°C, shaking at 250 rpm. After 5 days of growth, cultures were inoculated into fresh media and returned to the incubator for an additional 48 hours, resulting in 2 copies of each culture, 7 days old and 48 hours old. We adopted this procedure to account for variable growth rates of different SynCom members and to ensure that nonstationary cells from each strain were included in the inoculum. After growth, 48-hour-old and 7-day-old plates were combined and optical density (OD) of the culture was measured at 600 nm using an Infinite M200 Pro plate reader (TECAN, Männedorf, Switzerland). All cultures were then pooled while normalizing the volume of each culture according to the OD (we took a proportionally higher volume of culture from cultures with low OD). The mixed culture was then washed twice with 10 mM MgCl_2_ (MilliporeSigma, St. Louis, MO) to remove spent media and cell debris and vortexed vigorously with sterile glass beads to break up aggregates. OD of the mixed, washed culture was then measured and normalized to OD = 0.2. A total of 100 μL of this SynCom inoculum was spread on each agar plate prior to transferring seedlings.

#### Experimental design of agar experiments

We performed the Pi gradient experiment in 2 independent replicas (experiments performed at different times, with fresh bacterial inoculum and batch of plants), each containing 3 internal replications, amounting to 6 samples for each treatment. We had 2 SynCom treatments: no bacteria (NB) and SynCom; 6 Pi concentrations: 0, 10, 30, 50, 100, or 1,000 μM KH_2_PO_4_(henceforth, Pi); and 2 plant treatments: planted plates and unplanted plates (NP).

For the drop-out experiment, the entire SynCom, excluding all 5 *Burkholderia* and both *Ralstonia* isolates, was grown and prepared as described above. The *Burkholderia* and *Ralstonia* isolates were grown in separate tubes, washed, and added to the rest of the SynCom to a final OD of 0.001 (the calculated OD of each individual strain in a 185-Member SynCom at an OD of 0.2) to form the following 4 mixtures: (1) Full community—all *Burkholderia* and *Ralstonia* isolates added to the SynCom; (2) *Burkholderia* drop-out—only *Ralstonia* isolates added to the SynCom; (3) *Ralstonia* drop-out—only *Burkholderia* isolates added to the SynCom; (4) uninoculated plants—no SynCom. The experiment had 3 Pi conditions: low Pi (50 μM Pi), high Pi (1,000 μM Pi), and low→high Pi. Twelve days post-inoculation the low Pi and high Pi samples were harvested, and the low→high plants were transferred from 50 μM Pi plates to 1,000 μM Pi plates for an additional 3 days. The experiment was performed twice, and each rep consisted of 6 plates per SynCom mixture and Pi treatment, amounting to 72 samples. Upon harvest, shoot Pi accumulation was measured using the Ames method.

For the drop-out experiment with PSR mutants, the entire SynCom, excluding all 5 *Burkholderia*, was grown and prepared as described above. The *Burkholderia* isolates were grown in separate tubes, washed, and added to the SynCom to a final OD of 0.001 (the calculated OD of each individual strain in a 185-Member SynCom at an OD of 0.2) to form the following 2 mixtures: (1) Full community—all *Burkholderia* isolates added to the SynCom; (2) *Burkholderia* drop-out—no isolates added to the SynCom. For each SynCom, we inoculated 6 agar plates for each of 3 Pi conditions: 0, 50, and 1,000 μM Pi. Three 7-day-old seedlings from each of the 3 genotypes (wt Col-0, *phf1*, and *phr1* phl1) were transferred to each plate. Roots were harvested 12 days post-inoculation, and bacterial DNA was extracted.

#### In vitro plant growth conditions

*Arabidopsis thaliana* accession Col-0 was used. All seeds were surface-sterilized with 70% bleach (Clorox, Oakland, CA), 0.2% Tween-20 (MilliporeSigma, St. Louis, MO) for 8 minutes, and rinsed 3 times with sterile distilled water to eliminate any seed-borne microbes on the seed surface. Seeds were stratified at 4°C in the dark for 2 days. Plants were germinated on vertical square 12 X 12 cm agar plates (Fisher Scientific, Hampton, NH) with Johnson medium (JM; [4]) containing 0.5% sucrose (MilliporeSigma, St. Louis, MO) and 1,000 μM Pi, for 7 days. Then, 10 plants were transferred to each vertical agar plate with amended JM lacking sucrose at one of the following experimental Pi concentrations: 0, 10, 30, 50, 100, or 1,000 μM Pi. The SynCom was spread on the agar prior to transferring plants. Each experiment included unplanted agar plates with SynCom for each media type (designated NP) and uninoculated plates with plants for each media type (designated NB). Plants were placed in randomized order in growth chambers and grown under a 16-hour dark/8-hour light regime at 21°C day/18°C night for 12 days (the period of time it takes roots to reach the bottom of the plate).

#### Sample harvest

Twelve days post-transferring, plates were imaged using a document scanner. For DNA extraction, roots, shoots, and agar were harvested separately, pooling 6 plants for each sample. Roots and shoots were placed in 2.0 ml Eppendorf tubes with 3 sterile glass beads. Samples were washed 3 times with sterile distilled water to remove agar particles and weakly associated microbes. Tubes were stored at −80˚C until processing. For RNA, samples were collected from a separate set of 2 independent experiments, using the same SynCom and conditions as above but with just 2 Pi concentrations: 1,000 μM Pi (high) and 50 μM Pi (low). Four seedlings were harvested from each sample, and samples were flash frozen and stored at −80˚C until processing.

#### DNA extraction

Root and shoot samples were lyophilized for 48 hours using a Freezone 6 freeze dry system (Labconco, Fisher Scientific, Hampton, NH) and pulverized using a tissue homogenizer (MP Biomedicals, Solon, OH). Agar from each plate was stored in a 30 ml syringe (Fisher Scientific, Hampton, NH) with a square of sterilized Miracloth (Millipore) at the bottom and kept at −20°C for a week. Syringes were then thawed at room temperature, and samples were squeezed gently into 50 ml tubes. Samples were centrifuged at maximum speed for 20 minutes, and most of the supernatant was discarded. The remaining 1 to 2 ml of supernatant containing the pellet was transferred into clean microfuge tubes. Samples were centrifuged again, supernatant was removed, and pellets were stored at −80°C until DNA extraction.

DNA extractions were carried out on ground root and shoot tissue and agar pellets using 96-well-format MoBio PowerSoil Kit (MOBIO Laboratories; Qiagen, Hilden, Germany) following the manufacturer’s instruction. Sample position in the DNA extraction plates was randomized, and this randomized distribution was maintained throughout library preparation and sequencing.

#### RNA extraction

RNA was extracted from *Arabidopsis* seedlings following the work by Ames [61]. Frozen seedlings were ground in liquid nitrogen, then homogenized in a buffer containing 400 μl of Z6-buffer; 8 M guanidinium-HCl (MilliporeSigma, St. Louis, MO), 20 mM MES, (MilliporeSigma, St. Louis, MO)20 mM EDTA (MilliporeSigma, St. Louis, MO) at pH 7.0; 400 μL phenol:chloroform:isoamylalcohol (25:24:1) (MilliporeSigma, St. Louis, MO) was added, and samples were vortexed and centrifuged (20,000*g*, 10 minutes) for phase separation. The aqueous phase was transferred to a new 1.5 ml tube, and 0.05 volumes of 1 N acetic acid (MilliporeSigma, St. Louis, MO) and 0.7 volumes 96% ethanol were added. The RNA was precipitated at −20°C overnight. Following centrifugation (20,000*g*, 10 minutes, 4°C), the pellet was washed with 200 μl sodium acetate (pH 5.2) (MilliporeSigma, St. Louis, MO) and 70% ethanol. The RNA was dried and dissolved in 30 μL of ultrapure water and stored at −80°C until use.

#### Quantification of plant phenotypes

The Ames method [60] was used to determine the phosphate concentration in the shoots of plants grown on different Pi regimens and treatments. Primary root length elongation was measured using ImageJ [62], and for shoot area and total root network measurement, WinRhizo software (Regent Instruments Inc., Quebec, Canada) was used.

### DNA and RNA sequencing

#### Bacterial 16S sequencing

We amplified the V3-V4 regions of the bacterial 16S rRNA gene using primers 338F (5′-ACTCCTACGGGAGGCAGCA-3′) and 806R (5′-GGACTACHVGGGTWTCTAAT-3′). Two barcodes and 6 frames hifts were added to the 5’ end of 338F, and 6 frameshifts were added to the 806R primers, based on the protocol in the work by Lundberg and colleagues [63]. Each PCR reaction was performed in triplicate and included a unique mixture of 3 frameshifted primer combinations for each plate. PCR conditions were as follows: 5 μl Kapa Enhancer (Kapa Biosystems, Wilmington, MA), 5 μl Kapa Buffer A (Kapa Biosystems, Wilmington, MA), 1.25 μl of 5 μM 338F, 1.25 μl of 5 μM 806R, 0.375 μl mixed rRNA gene-blocking peptide nucleic acids (PNAs; 1:1 mix of 100 μM plastid PNA and 100 μM mitochondrial PNA; PNA Bio (Kapa Biosystems, Wilmington, MA), 0.5 μl Kapa dNTPs (Kapa Biosystems, Wilmington, MA), 0.2 μl Kapa Robust Taq (Kapa Biosystems, Wilmington, MA), 8 μl dH2O, 5 μl DNA; temperature cycling: 95°C for 60 seconds, 24 cycles of 95°C for 15 seconds, 78°C (PNA) for 10 seconds, 50°C for 30 seconds, 72°C for 30 seconds, 4°C until use. Following PCR cleanup, the PCR product was indexed using 96 indexed 806R primers with the same reaction mix as above and 9 cycles of the cycling conditions described in the work by Lundberg and colleagues [63]. PCR products were purified using AMPure XP magnetic beads (Beckman Coulter, Brea, CA) and quantified with a Qubit 2.0 fluorometer (Invitrogen, Carlsbad, CA). Amplicons were pooled in equal amounts and then diluted to 10 pM for sequencing. Sequencing was performed on an Illumina MiSeq instrument (Illumina, San Diego, CA) using a 600-cycle V3 chemistry kit. The raw data for the natural soil experiment is available in the NCBI SRA Sequence Read Archive (accession PRJNA531340). The raw data for the SynCom experiment is available in the NCBI SRA Sequence Read Archive (accession PRJNA531340).

#### Fungal/Oomycete ITS sequencing

We amplified the ITS1 region using primers ITS1-F (5′-CTTGGTCATTTAGAGGAAGTAA-3′; [64]) and ITS2 (5′-GCTGCGTTCTTCATCGATGC-3′; [65]). Samples were diluted to concentrations of 3.5 ng μl^−1^ of DNA with nuclease-free water for the first PCR reaction to amplify the ITS1 region. Reactions were prepared in triplicate in 25 μl volumes consisting of 10 ng of DNA template, 1× incomplete buffer, 0.3% bovine serum albumin, 2 mM MgCl_2_, 200 μM dNTPs, 300 nM of each primer, and 2 U of DFS-Taq DNA polymerase (Bioron, Ludwigshafen, Germany); temperature cycling: 2 minutes at 94°C, 25 cycles: 30 seconds at 94°C, 30 seconds at 55°C, and 30 seconds at 72°C; and termination: 10 minutes at 72°C. PCR products were cleaned using an enzymatic cleanup (24.44 μl: 20 μl of template, 20 U of exonuclease I, 5 U of Antarctic phosphatase, 1× Antarctic phosphatase buffer; New England Biolabs, Frankfurt, Germany); incubation conditions were 30 minutes at 37°C, 15 minutes at 85°C; centrifuge 10 minutes at 4,000 rpm. A second PCR was then performed (2 minutes at 94°C; 10 cycles: 30 seconds at 94°C, 30 seconds at 55°C, and 30 seconds at 72°C; and termination: 10 minutes at 72°C), in triplicate using 3 μl of cleaned PCR product and sample-specific barcoded primers (5′- AATGATACGGCGACCACCGAGATCTACACTCACGCGCAGG-ITS1F-3′; 5′-CAAGCAGAAGACGGCATACGAGAT-BARCODE(12-NT)-CGTACTGTGGAGA-ITS2-3′). PCR reactions were purified using with Agencourt AMPure XP purification kit (Beckman Coulter, Krefeld, Germany). Amplicons were pooled in equal amounts and then diluted to 10 pM for sequencing. Sequencing was performed on an Illumina MiSeq instrument using a 600-cycle V3 chemistry kit. The raw data are available in the NCBI SRA Sequence Read Archive (Project Number PRJNA531340).

#### Plant RNA sequencing

Illumina-based mRNA-Seq libraries were prepared from 1 μg RNA following the work by Herrera Paredes and colleagues [38]. mRNA was purified from total RNA using Sera-mag oligo(dT) magnetic beads (GE Healthcare Life Sciences, Chicago, IL) and then fragmented in the presence of divalent cations (Mg^2+^) at 94°C for 6 minutes. The resulting fragmented mRNA was used for first-strand cDNA synthesis using random hexamers and reverse transcriptase (Enzymatics, Qiagen, Beverly, MA), followed by second-strand cDNA synthesis using DNA Polymerase I (Enzymatics, Qiagen, Beverly, MA) and RNAseH (Enzymatics, Qiagen, Beverly, MA). Double-stranded cDNA was end-repaired using T4 DNA polymerase (Enzymatics, Qiagen, Beverly, MA), T4 polynucleotide kinase (Enzymatics, Qiagen, Beverly, MA), and Klenow polymerase (Enzymatics, Qiagen, Beverly, MA). The DNA fragments were then adenylated using Klenow exo-polymerase (Enzymatics, Qiagen, Beverly, MA) to allow the ligation of Illumina Truseq HT adapters (D501–D508 and D701–D712; Illumina, San Diego, CA). Following library preparation, quality control and quantification were performed using a 2100 Bioanalyzer instrument (Agilent Technologies, Santa Clara, CA) and the Quant-iT PicoGreen dsDNA Reagent (Invitrogen, Carlsbad, CA), respectively. Libraries were sequenced using HiSeq4000 sequencers (Illumina, San Diego, CA) to generate 50-bp single-end reads.

### Data processing and statistical analyses

#### Quantification of plant phenotypes—Soil experiment

To measure correlation between all measured plant phenotypes (shoot Pi, shoot weight, shoot size) we applied hierarchical clustering based on a matrix of Pearson correlation coefficients between all pairs of phenotypes. We used the R package corrplot version 0.84 [66] to visualize correlations. To compare shoot Pi accumulation, we treated the low P sample as the control, because this soil did not receive any treatment. We performed paired *t* tests between the different P-treated samples and the low P samples within each plant genotype independently (α < 0.05).

#### Amplicon sequence data processing—Soil experiments

Bacterial sequencing data were processed with MT-Toolbox [67]. Usable read output from MT-Toolbox (i.e., reads with 100% correct primer and primer sequences that successfully merged with their pair) were quality filtered using Sickle [68] by not allowing any window with a Q score under 20. After quality filtering, samples with low total reads recruited (<3,000 reads), amounting to 51 soil samples were discarded. Although this study focuses on the root microbiome, the relatively small number of remaining soil samples may have affected the results shown in S4E Fig. The remaining samples include at least 3 samples per genotype. The resulting sequences were collapsed into ASVs using the R package DADA2 version 1.8.1 [69]. Taxonomic assignment of each ASV was performed using the naïve Bayes kmer method implemented in the DADA2 package using the Silva 132 database as training reference [69].

Fungal ITS sequence data were processed using DADA2 [69] with default parameters using only the forward reads. Taxonomic assignment of each ASV was performed using the naïve Bayes kmer method implemented in the MOTHUR package [70] using the UNITE database [71] as training reference.

The resulting bacterial and fungal count tables were deposited at https://github.com/isaisg/hallepi.

#### Community analyses—Soil experiments

The resulting bacterial and fungal count tables were processed and analyzed with functions from the ohchibi package [72]. Both tables were rarefied to 3,000 reads per sample. An alpha diversity metric (Shannon diversity) was calculated using the diversity function from the vegan package version 2.5–3 [73]. We used ANOVA to test for differences in Shannon Diversity indices between groups. Tukey’s HSD post hoc tests here and elsewhere were performed using the cld function from the emmeans R package [74]. Beta-diversity analyses (Principal coordinate analysis and canonical analysis of principal coordinates [CAP]) were based on Bray-Curtis dissimilarity calculated from the rarefied abundance tables. We utilized the capscale function from the vegan R package v.2.5–3 [73] to compute a CAP. To analyze the full data set (all fraction, all genotypes, all phosphorus treatments), we constrained by fraction, plant genotype, and phosphorus fertilization treatment, while conditioning for the plot effect. We performed the genotype:phosphorus interaction analysis over each fraction independently, constraining for the plant genotype and phosphorus fertilization treatment while conditioning for the plot effect. In addition to CAP, we performed Permutational Multivariate Analysis of Variance (PERMANOVA) over the 2 data sets described above using the adonis function from the vegan package version 2.5–3 [73]. Finally, we used the function chibi.permanova from the ohchibi package to plot the R^2^ values for each significant term in the PERMANOVA model tested.

The relative abundance of bacterial phyla and fungal taxa were depicted using the stacked bar representation encoded in the function chibi.phylogram from the ohchibi package.

We used the R package DESeq2 version 1.22.1 [75] to compute the enrichment profiles for both bacterial and fungal ASVs. For the full data set model, we estimated main effects for each variable tested (Fraction, Plant genotype, and phosphorus fertilization) using the following design:

Abundance ~ Fraction + Genotype + Phosphorus Treatment

We delimited ASV fraction enrichments using the following contrasts: soil versus root, soil versus shoot, and root versus shoot. An ASV was considered statistically significant if it had *q* < 0.1.

We implemented a second statistical model in order to identify ASVs that exhibited statistically significant differential abundances depending on genotype. For this analysis, we utilized only root-derived low P and P-supplemented low P (low+P) treatments. We utilized a group design framework to facilitate the construction of specific contrasts. In the group variable we created, we merged the genotype and phosphate levels per sample (e.g., Col-0_lowP, *phf1*_low+P, or *phr1 phl1*_lowP). We controlled the paired structure of our design by adding a plot variable, resulting in the following model design:

Abundance ~ Plot + group

We delimited 6 sets (S1, S2, S3, S4, S5, S6) of statistically significant (*q* < 0.1) ASVs from our model using the following contrasts:

*S1* = {Samples from Col-0, higher abundance in low treatment in comparison to low+P treatment}
*S2* = {Samples from *phf1*, higher abundance in low treatment in comparison to low+P treatment}
*S3* = {Samples from *phr1 phl1*, higher abundance in low treatment in comparison to low+P treatment}
*S4* = {Samples from *Col-0*, higher abundance in low+P treatment in comparison to low treatment}
*S5* = {Samples from *phf1*, higher abundance in low+P treatment in comparison to low treatment}
*S6* = {Samples from *phr1 phl1*, higher abundance in low+P treatment in comparison to low treatment}

The 6 sets described above were used to populate Fig 3C–3F.

The interactive visualization of the enrichment profiles was performed by converting the taxonomic assignment of each ASV into a cladogram with equidistant branch lengths using R. We used the interactive tree of life (iTOL) interface [76] to visualize this tree jointly with metadata files derived from the output of the statistical models described above. The cladograms for both bacteria and fungi can be downloaded from the links described above or via the iTOL user hallepi.

In order to compare beta-diversity patterns across samples, we only used samples coming from pots in which sequence data from all 3 fractions (soil, root, and shoot) passed quality filtering. Then, for each fraction, we estimated a distance structure between samples inside that fraction using the Bray-Curtis dissimilarity metric. Finally, we computed Mantel [77] correlations between pairs of distance objects (e.g., samples from root or samples from shoot) using the vegan package version 2.5–3 [73] implementation of the Mantel test.

All scripts and data sets required to reproduce the soil experiment analyses are deposited in the following GitHub repository: https://github.com/isaisg/hallepi/.

#### Inspection of other edaphic factors in the soil

To inspect whether the genotype or P effects that we observed are confounded by another edaphic factor in the soil, we cross-referenced our data set with the edaphic factors reported for the same soil plots in the work by Robbins and colleagues [43]. Because most of the edaphic factors are correlated (S5A Fig), we considered the first 3 principal components (PCs) derived from these edaphic factors. These 3 PCs encompass 87% of the cumulative variance in the edaphic factor matrix (S5B Fig). A PERMANOVA model of the root community composition that considers these 3 PCs assigns 21% of explained variance to the first PC, which is composed of 8 edaphic factors (S5C and S5B Fig). Nonetheless, the P and genotype variables explain a similar proportion of variance as in a model that did not account for the other edaphic factors, indicating that they are orthogonal to the other variables that can be accounted for and are not confounded by them.

#### Amplicon sequence data processing—SynCom experiments

SynCom sequencing data were processed with MT-Toolbox [67]. Usable read output from MT-Toolbox (i.e., reads with 100% correct primer and primer sequences that successfully merged with their pair) were quality filtered using Sickle [68] by not allowing any window with Q-score under 20. The resulting sequences were globally aligned to a reference set of 16S rRNA gene sequences extracted from genome assemblies of SynCom member strains. For strains that did not have an intact 16S rRNA gene sequence in their assembly, we generated the 16S rRNA gene using Sanger sequencing. The reference database also included sequences from known bacterial contaminants and *Arabidopsis* organellar 16S sequences (S12 Table). Sequence alignment was performed with USEARCH version 7.1090 [78] with the option ‘usearch_global’ at a 98% identity threshold. On average, 85% of sequences matched an expected isolate. Our 185 isolates could not all be distinguished from each other based on the V3-V4 sequence and were thus classified into 97 USeqs. A USeq encompasses a set of identical (clustered at 100%) 16S rRNA V3-V4 sequences coming from a single or multiple isolates.

Sequence mapping results were used to produce an isolate abundance table. The remaining unmapped sequences were clustered into Operational Taxonomic Units (OTUs) using UPARSE [79] implemented with USEARCH version 7.1090 at 97% identity. Representative OTU sequences were taxonomically annotated with the RDP classifier [80] trained on the Greengenes database [81] (4 February 2011). Matches to *Arabidopsis* organelles were discarded. The vast majority of the remaining unassigned OTUs belonged to the same families as isolates in the SynCom. We combined the assigned USeq and unassigned OTU count tables into a single table.

The resulting count table was processed and analyzed with functions from the ohchibi package. Samples were rarefied to 1,000 reads per sample. An alpha diversity metric (Shannon diversity) was calculated using the diversity function from the vegan package version 2.5–3 [73]. We used ANOVA to test for differences in alpha diversity between groups. Beta-diversity analyses (Principal coordinate analysis and CAP) were based on were based on Bray-Curtis dissimilarity calculated from the rarefied abundance tables. We used the capscale function from the vegan R package version 2.5–3 [73] to compute the CAP. To analyze the full data set (all fraction, all phosphate treatments), we constrained by fraction and phosphate concentration while conditioning for the replicate effect. We performed the Fraction:Phosphate interaction analysis within each fraction independently, constraining for the phosphate concentration while conditioning for the rep effect. In addition to CAP, we used PERMANOVA analysis over the 2 data sets described above using the adonis function from the vegan package version 2.5–3 [73]. Finally, we used the function chibi.permanova from the ohchibi package to plot the R^2^ values for each significant term in the PERMANOVA model tested.

We visualized the relative abundance of the bacterial phyla present in the SynCom using the stacked bar representation encoded in the chibi.phylogram from the ohchibi package.

We used the package DESeq2 version 1.22.1 [75] to compute the enrichment profiles for USeqs and OTUs present in the count table. For the full data set model, we estimated main effects for each variable tested (fraction and phosphate concentration) using the following model specification:

Abundance ~ Fraction + Phosphate Treatment + Replicate

We calculated the USeqs/OTUs fraction enrichments using the following contrasts: agar versus root, agar versus shoot, and root versus shoot. A USeq/OTU was considered statistically significant if it had *q* < 0.1. In order to populate the heat maps shown in Fig 5C, we grouped the fraction and phosphate treatment variable into a new group variable that allowed us to fit the following model:

Abundance ~ Replicate + group

We used the fitted model to estimate the fraction effect inside each particular phosphate level (e.g., Root versus agar at 0Pi, or shoot versus agar at 1,000Pi).

Additionally, we utilized a third model for the identification of USeqs/OTUs that exhibited a significant Fraction:Phosphate interaction between the planted agar samples and the plant fractions (root and shoot). Based on the beta-diversity and alpha-diversity results, we only used samples that were treated with 0, 10, 100, and 1,000 μM of phosphate. We grouped the samples into 2 categories based on their phosphate concentration, low (0 μM and 10 μM) and high (100 μM and 1,000 μM). Then we used the following model specification to derive the desired interaction effect:

Abundance ~ Fraction + Category + Fraction:Category + Replicate

Finally, we subset USeqs that exhibited a significant interaction (Fraction:Category, *q* < 0.1) in the following 2 contrasts (planted agar versus root) and (planted agar versus shoot).

In order to compare beta-diversity patterns across samples, we only used samples coming from pots in which sequence data from all 3 fractions (soil root and shoot) passed quality filtering. Then, for each fraction, we estimated a distance structure between samples inside that fraction using the Bray-Curtis dissimilarity metric. Finally, we computed Mantel [77] correlations between pairs of distance objects (e.g., samples from root or samples from shoot) using the vegan package version 2.5–3 [73] implementation of the Mantel test.

For the drop-out experiment, we ran an ANOVA model inside each of the phosphate treatments tested (50 μM Pi, 1,000 μM Pi, and 50→1,000 μM Pi). We visualized the results of the ANOVA models using the compact letter display encoded in the CLD function from the emmeans package.

All scripts necessary to reproduce the synthetic community analyses are deposited in the following GitHub repository: https://github.com/isaisg/hallepi.

#### Phylogenetic inference of the SynCom isolates

To build the phylogenetic tree of the SynCom isolates, we utilized the supermatrix approach previously described in the work by Levy and colleagues [50]. Briefly, we scanned 120 previously defined marker genes across the 185 isolate genomes from the SynCom utilizing the hmmsearch tool from the hmmer version 3.1b2 [82]. Then, we selected 47 markers that were present as single copy genes in 100% of our isolates. Next, we aligned each individual marker using MAFFT [83] and filtered low quality columns in the alignment using trimAl [84]. Afterward, we concatenated all filtered alignments into a superalignment. Finally, FastTree version 2.1 [85] was used to infer the phylogeny utilizing the WAG model of evolution.

We utilized the inferred phylogeny along with the fraction fold change results of the main effect model to compute the phylogenetic signal (Pagel’s λ) [86] for each contrast (planted agar versus root, planted agar versus shoot, and root versus shoot) along each concentration of the phosphate gradient. The function phylosig from the R package phytools [87] was used to test for significance of the phylogenetic signal measured.

Multiple panel figures were constructed using the egg R package [88].

#### RNA-Seq read processing

Initial quality assessment of the Illumina RNA-Seq reads was performed using FastQC version 0.11.7 [89]. Trimmomatic version 0.36 [90] was used to identify and discard reads containing the Illumina adaptor sequence. The resulting high-quality reads were then mapped against the TAIR10 [91] *Arabidopsis* reference genome using HISAT2 version 2.1.0 [92] with default parameters. The featureCounts function from the Subread package [93] was then used to count reads that mapped to each one of the 27,206 nuclear protein-coding genes. Evaluation of the results of each step of the analysis was done with MultiQC version 1.1 [94]. Raw sequencing data and read counts are available at the NCBI Gene Expression Omnibus accession number GSE129396.

#### RNA-Seq statistical analysis—Soil experiment

To measure the transcriptional response to Pi limitation in soil, we used the package DESeq2 version 1.22.1 [75] to define differentially expressed genes (DEGs) using the raw count table described above. We used only samples from low P and P-supplemented low P (low+P) treatments along the 3 genotypes tested (Col-0, *phf1*, and *phr1 phl1*). We combined the genotype and P treatment variables into a new group variable (e.g., Col-0_lowP or *phf1*_low+P). Because we were interested in identifying DEGs among any pair of levels (6 levels) of the group variable (e.g., Col-0_lowP versus Col-0_low+P) we performed a likelihood ratio test (LRT) between a model containing the group variable and a reduced model containing only the intercept. Next, we defined DEGs as genes that had a *q* < 0.1.

For visualization purposes, we applied a variance stabilizing transformation to the raw count gene matrix. We then standardized (z-score) each gene along the samples. We subset DEGs from this standardized matrix and for each gene calculated the mean z-score expression value in a particular level of the group variable (e.g., Col-0_lowP); this resulted in a matrix of DEGs across the 6 levels in our design. Next, we created a dendrogram of DEGs by applying hierarchical clustering (method ward.D2, hclust R-base [95]) to a distance object based on the correlation (dissimilarity) of the expression profiles of the genes across the 6 levels in our design. Finally, we delimited the cluster of DEGs by cutting the output dendogram into 5 groups using the R-base cutree function [95]. GO enrichment was performed for each cluster of DEGs using the R package clusterProfiler [96].

For the PSR marker gene analysis, we downloaded the ID of 193 genes defined in the work by Castrillo and colleagues [4]. Then, we subset these genes from our standardized matrix and computed for each gene the mean z-score expression value in a particular level of the group variable. Finally, we visualized the average expression of this PSR regulon across our groups of interest utilizing the function chibi.boxplot from the ohchibi package.

All scripts necessary to reproduce the RNA-Seq analyses are deposited in the following GitHub repository: https://github.com/isaisg/hallepi.

#### RNA-Seq statistical analysis—SynCom experiment

To measure the transcriptional response to Pi limitation in the SynCom microcosm, we used the package DESeq2 version 1.22.1 [75] to define DEGs using the raw count gene table. We combined the bacteria (NB, Full SynCom) and P treatment variables into a new group variable (e.g., NB_50Pi or Full_1000Pi). Afterward we fitted the following model to our gene matrix:

Abundance Gene ~ Rep + group

Finally, utilizing the model fitted, we contrasted the phosphate treatment inside each level of the bacteria variable (e.g., NB_1000Pi versus NB_50Pi). Any gene with *q* < 0.1 was defined as differentially expressed.

For the PSR marker gene analysis, we downloaded the ID of 193 genes defined in the work by Castrillo and colleagues [4]. Then, we subset these genes from our standardized matrix and computed for each gene the mean z-score expression value in a particular level of the group variable. Finally, we visualized the average expression of the PSR regulon across our groups of interest utilizing the function chibi.boxplot from the ohchibi package.

All scripts necessary to reproduce the RNA-Seq analyses are deposited in the following GitHub repository: https://github.com/isaisg/hallepi.

## Supporting information

S1 Fig PSR in soil. (A) Heat map showing the all versus all pairwise Pearson correlation coefficient calculated between the quantified phenotypes associated with the PSR: shoot area, shoot fresh weight, and shoot free Pi accumulation. (B) Box plot showing the distribution of the shoot area measured across the P gradient within each of the 3 genotypes. (C) Boxplot showing the distribution of shoot fresh weight measured across the P gradient within each of the 3 genotypes. (D) Box plot showing the shoot Pi accumulation across the 3 genotypes. Letters represent the results of the post hoc test. (E) Box plots displaying the average expression of 193 PSR marker genes across the low and low+P samples in each of the 3 genotypes tested. (F) Scatter plot showing the relationship between the standardized average phosphate accumulation in leaves (x-axis) and the average standardized expression of 193 PSR marker genes (y-axis). The *p*-value and R value were calculated according to Pearson’s product moment correlation coefficient. P, phosphorus; Pi, orthophosphate; PSR, phosphate starvation response.
(TIF)

S2 Fig Characterization of the soil and plant microbiota in soils exposed to different level of P fertilization. (A, B) Box plots showing the distribution of the alpha diversity (Shannon diversity index) across all levels of P in the soil for bacteria (A) and fungi (B). (C, D) PERMANOVA results in which the effect of the 3 variables (fraction, genotype, and soil P) and their interaction on the assembly of the bacterial (C) and fungal (D) communities were tested. (E) Relative abundance profiles of the main bacterial phyla in the 3 variables (fraction, genotype, and soil P) across all levels of P in the soils. (F) Relative abundance profiles of the main fungal orders in the 3 variables (fraction, genotype, and soil P) across all the levels of P in the soils. P, phosphorus; PERMANOVA, Permutational Multivariate Analysis of Variance.
(TIF)

S3 Fig Bacterial but not fungal plant microbiota composition is strongly dependent on soil inoculum. (A, B, C) Correlation plots between Bray-Curtis distance matrices calculated for bacteria within soil treatments, root, and shoot fractions. The R and *p*-values were calculated using Mantel tests. (A) Correlation plot of soil versus root. (B) Correlation plot of soil versus shoot. (C) Correlation plot of root versus shoot. (D, E, F) Correlation plots between Bray-Curtis distance matrices calculated for fungi within soil treatments, root, and shoot fractions. The R and *p*-values were calculated using Mantel tests. R and *p*-values colored in red were calculated excluding the cloud of large distances appearing in graphs panels D and E. (D) Correlation plot of soil versus root. (E) Correlation plot of soil versus shoot. (F) Correlation plot of root versus shoot.
(TIF)

S4 Fig Plant genotypes and soil P concentrations influence the composition of the plant microbiota. (A, B) PERMANOVA results showing the influence of the plant genotype and soil P concentration and their interaction on the assembly of the root (A) bacterial and (B) fungal communities. (C, D) CAP showing the effect of plant genotype and P content in the soil over the shoot (C) bacterial and (D) fungal communities. The *p*-value and R^2^ values in each plot are derived from a PERMANOVA model and correspond to the genotype and soil P term, respectively. (E, F) CAP showing the influence of genotype and P on the soil (E) bacterial and (F) fungal communities. Note smaller number of points in bacterial soil samples. The *p*-value and R^2^ values in each plot are derived from a PERMANOVA model and correspond to the genotype and soil P term, respectively. CAP, canonical analysis of principal coordinates; P, phosphorus; PERMANOVA, Permutational Multivariate Analysis of Variance.
(TIF)

S5 Fig Variation in soil edaphic factors does not confound soil P effect. (A) Correlation heat map of the 13 edaphic factors reported in the work by Robbins and colleagues [43]. (B) Bar plot displaying the amount of variance in the edaphic factor matrix explained by PC. (C) Bar plot depicting the contribution of the different edaphic factors to the first 3 PC. Colors denote the direction of the variable in PCA space. (D) Proportion of the variance explained for the different variables in models including (M2) and excluding (M1) edaphic factors for bacteria (left) and fungi (right). Only variables with a statistically significant effect are shown. P, phosphorus; PC, principal component; PCA, principal component analysis.
(TIF)

S6 Fig A bacterial synthetic community modifies the plant PSR. (A) Box plots displaying the primary root elongation of plants grown in a gradient of Pi concentrations in sterile conditions or with the SynCom. A *t* test was used for each Pi treatment to estimate differences between SynCom-treated and uninoculated plants. (B) Average expression of the 193 PSR markers genes in low (50 μM) and high (1,000 μM) Pi conditions within SynCom-treated and uninoculated plants. (C) Average expression of the 123 genes from Cluster 1 (Fig 1B) in low (50 μM) and high (1,000 μM) Pi conditions within SynCom-treated and uninoculated plants. (D) PERMANOVA model results showing the influence of the 2 variables (fraction and Pi concentration) and their interaction on the assembly of the bacterial community in the plant. (E, F, G) Correlation plots between Bray-Curtis distance matrices calculated for bacterial profile within agar, root, and shoot fractions. The R and *p*-values were calculated using Mantel tests. (E) Correlation plot of agar versus root. (F) Correlation plot of agar versus shoot. (G) Correlation plot of root versus shoot. PERMANOVA, Permutational Multivariate Analysis of Variance; Pi, orthophosphate; PSR, phosphate starvation response; SynCom, synthetic community.
(TIF)

S7 Fig Bacterial synthetic community responds to the phosphate concentration in the media. (A, B) CAP showing the influence of Pi concentration in the media on the bacterial communities in the (A) plant shoot and (B) agar. The bar graphs to the left of each plot depict the percentage of variability explained by statistically significant (*p* < 0.05) variables based on a PERMANOVA model. (C, D, E) PERMANOVA model results showing the influence of Pi concentration on the assembly of the bacterial community in (C) roots, (D) shoot, and (E) agar. CAP, canonical analysis of principal coordinates; PERMANOVA, Permutational Multivariate Analysis of Variance; Pi, orthophosphate.
(TIF)

S8 Fig USeqs in the bacterial synthetic community displayed a strong Pi:fraction (shoot, root, agar) interaction. (A) Scatter plot (volcano plot) showing the results of the GLM interaction model between fraction and Pi concentration. The axes of the plot represent the output of the statistical test. The x-axis is the transformed *q*-value and the y-axis the log_2_ fold change. Each dot in the scatter plot represents a USeq. USeqs that showed a statistically significant fraction:Pi interaction are colored in red. USeqs genus and ID are displayed. The top right quadrant represents USeqs that are enriched in the plant tissues under low Pi conditions. (B, C) Relative abundance of *Burkholderia* Useqs 16 (B) and 30 (C) that exhibits a statistically significant (*q* < 0.1) Pi enrichment between the plant fractions and the agar fraction. The middle dot of each strip bar corresponds to the mean of that particular condition; the range of the strip bar corresponds to the 95% confidence interval of the mean. GLM, generalized linear model; Pi, orthophosphate; USeq, unique sequence.
(TIF)

S9 Fig Shoot size is not affected by *Burkholderia* drop-out. Box plots showing shoot circumference in plants exposed to different SynComs across 3 phosphate treatments. Statistically significant differences among SynCom treatments were computed within each phosphate treatment separately using an ANOVA model. Letters represent the results of the post hoc test. SynCom, synthetic community.
(TIF)

S10 Fig Plant genotype and Pi concentration affect root community composition in the agar system. CAP showing the influence of plant genotypes (A) and agar Pi concentration (B) over the bacterial SynCom in the root. The *p*-value and R^2^ values inside each plot are derived from a PERMANOVA model and correspond to the genotype and Pi terms, respectively. CAP, canonical analysis of principal coordinates; PERMANOVA, Permutational Multivariate Analysis of Variance; Pi, orthophosphate; SynCom, synthetic community.
(TIF)

S1 Table Shoot Pi concentration, weight, and size. Pi, orthophosphate.
(XLSX)

S2 Table Summary of RNA-Seq results presented in Fig 1. RNA-Seq, RNA sequencing.
(XLSX)

S3 Table Alpha diversity data for the soil experiment. (XLSX)

S4 Table Results of the negative binomial GLM testing the fraction (root, shoot, soil) effect on the bacterial ASVs). ASV, amplicon sequence variant; GLM, generalized linear model.
(XLSX)

S5 Table Results of the negative binomial GLM testing the fraction (root, shoot, soil) effect on the fungal ASVs. ASV, amplicon sequence variant; GLM, generalized linear model.
(XLSX)

S6 Table Results of the negative binomial GLM testing the Pi effect (low+P versus low) and the genotype effect within low and low+P on the bacterial ASVs. ASV, amplicon sequence variant; GLM, generalized linear model; P, phosphorus; Pi, orthophosphate.
(XLSX)

S7 Table Results of the negative binomial GLM testing the Pi effect (low+P versus low) and the genotype effect within low and low+P on the fungal ASVs. ASV, amplicon sequence variant; GLM, generalized linear model; P, phosphorus; Pi, orthophosphate.
(XLSX)

S8 Table Map of USeqs to bacterial strains in culture collections and their genome IDs. USeq, unique sequence.
(XLSX)

S9 Table Alpha diversity data for the agar experiment. (XLSX)

S10 Table Results of the negative binomial GLM in the agar system. GLM, generalized linear model.
(XLSX)

S11 Table Results for the Fraction:Pi interaction term in the negative binomial GLM for the agar system. GLM, generalized linear model; Pi, orthophosphate.
(XLSX)

S12 Table List of known contaminant 16S rRNA sequences. (XLSX)

S1 Data Folder containing individual files with underlying data for all figures presented in this paper in CSV format. (ZIP)

## Abbreviations

ASV: amplicon sequence variant
CAP: canonical analysis of principal coordinates
DEG: differentially expressed gene
GLM: generalized linear model
GO: gene ontology
HSD: honestly significant difference
iTOL: interactive tree of life
JM: Johnson medium
LRT: likelihood ratio test
NB: no bacteria
NP: unplanted plates
OD: optical density
OTU: Operational Taxonomic Unit
P: phosphorus
PC: principal component
PERMANOVA: Permutational Multivariate Analysis of Variance
PHF1: PHOSPHATE TRANSPORTER TRAFFIC FACILITATOR1
PHL1: PHR1-LIKE; PHR1, PHOSPHATE STARVATION RESPONSE 1
Pi: orthophosphate
PNA: peptide nucleic acid
PSR: phosphate starvation response
RNA-Seq: RNA sequencing
SynCom: synthetic community
USeq: unique sequence
wt: wild type
