# Supplementary figures and images for: The effects of soil phosphorus content on plant microbiota are driven by the plant phosphate starvation response
Source: PLoS Biol. 2019 Nov 13;17(11):e3000534. doi: 10.1371/journal.pbio.3000534 (PMC6876890; doi:10.1371/journal.pbio.3000534)

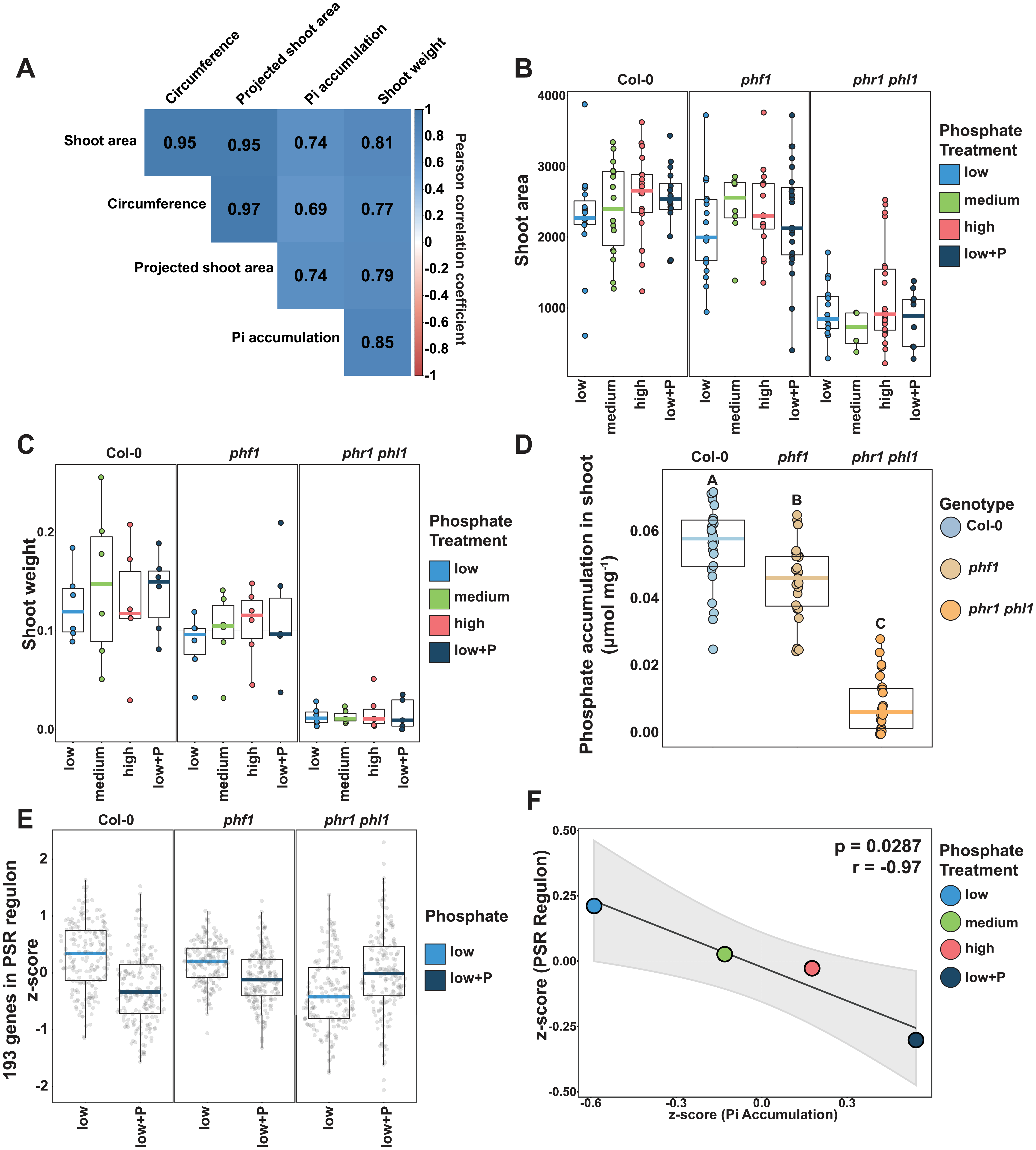

Supplement: S1 Fig — (A) Heat map showing the all versus all pairwise Pearson correlation coefficient calculated between the quantified phenotypes associated with the PSR: shoot area, shoot fresh weight, and shoot free Pi accumulation. (B) Box plot showing the distribution of the shoot area measured across the P gradient within each of the 3 genotypes. (C) Boxplot showing the distribution of shoot fresh weight measured across the P gradient within each of the 3 genotypes. (D) Box plot showing the shoot Pi accumulation across the 3 genotypes. Letters represent the results of the post hoc test. (E) Box plots displaying the average expression of 193 PSR marker genes across the low and low+P samples in each of the 3 genotypes tested. (F) Scatter plot showing the relationship between the standardized average phosphate accumulation in leaves (x-axis) and the average standardized expression of 193 PSR marker genes (y-axis). The p-value and R value were calculated according to Pearson’s product moment correlation coefficient. P, phosphorus; Pi, orthophosphate; PSR, phosphate starvation response. (TIF) [file pbio.3000534.s001.tif]

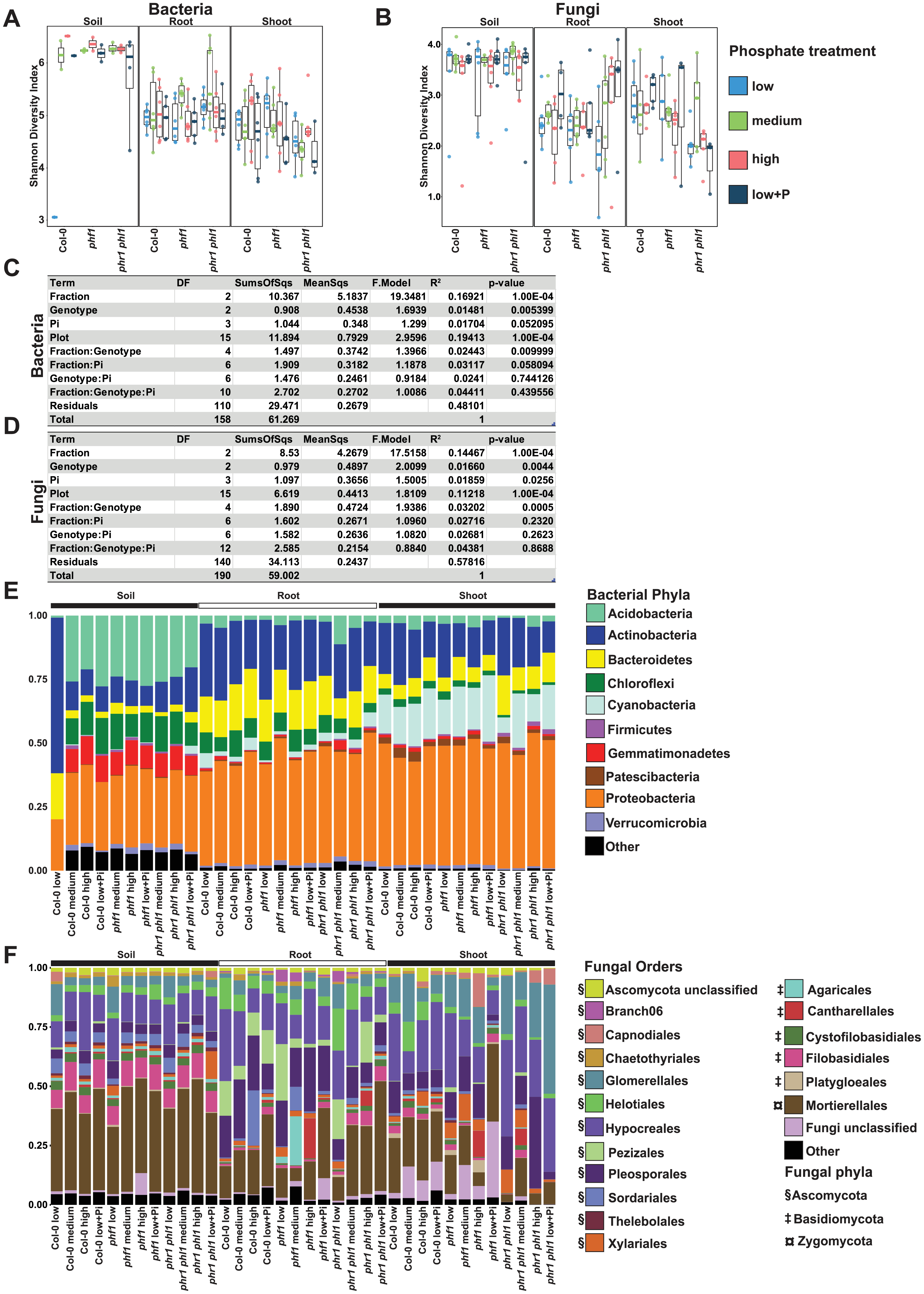

Supplement: S2 Fig — (A, B) Box plots showing the distribution of the alpha diversity (Shannon diversity index) across all levels of P in the soil for bacteria (A) and fungi (B). (C, D) PERMANOVA results in which the effect of the 3 variables (fraction, genotype, and soil P) and their interaction on the assembly of the bacterial (C) and fungal (D) communities were tested. (E) Relative abundance profiles of the main bacterial phyla in the 3 variables (fraction, genotype, and soil P) across all levels of P in the soils. (F) Relative abundance profiles of the main fungal orders in the 3 variables (fraction, genotype, and soil P) across all the levels of P in the soils. P, phosphorus; PERMANOVA, Permutational Multivariate Analysis of Variance. (TIF) [file pbio.3000534.s002.tif]

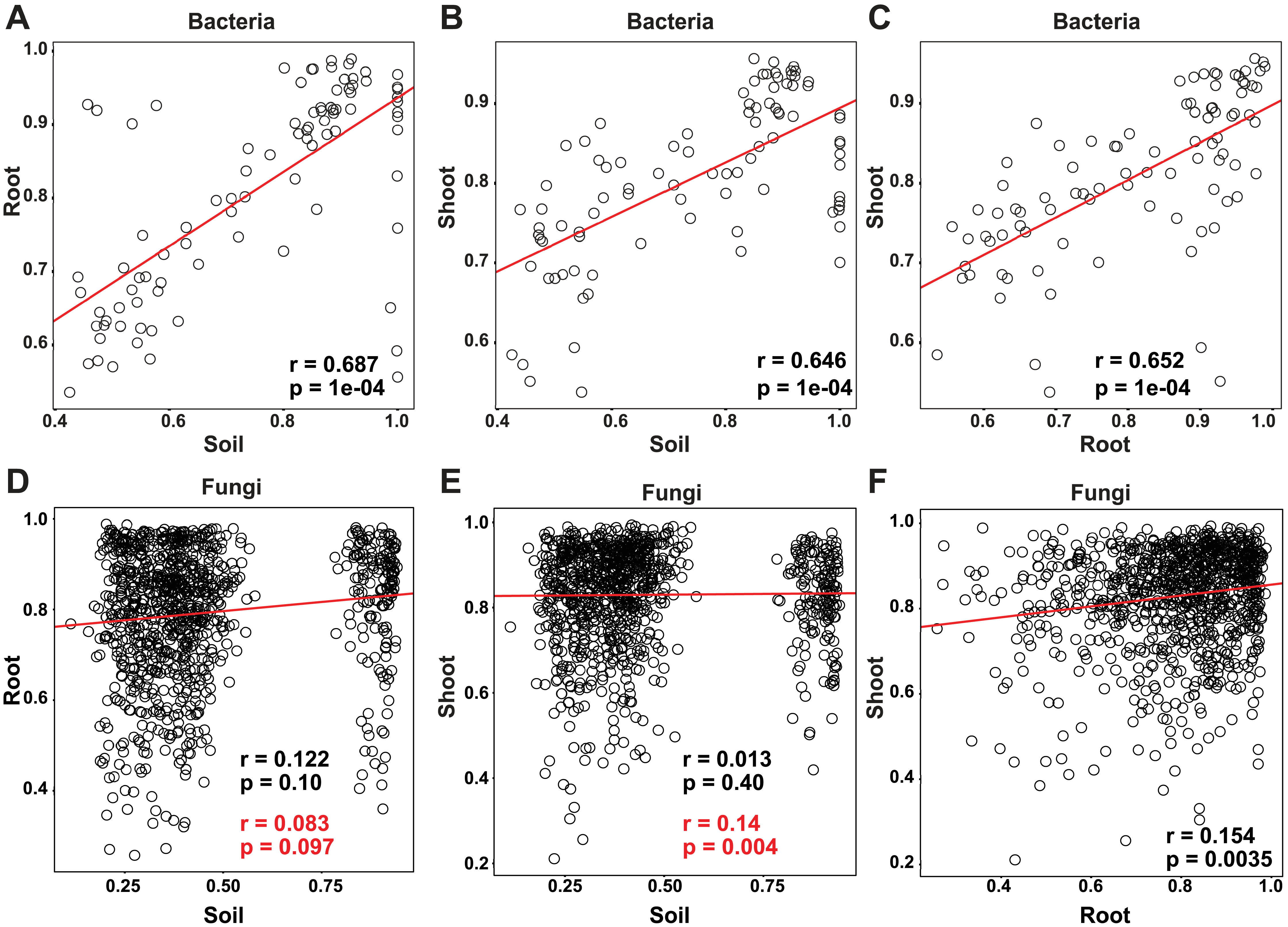

Supplement: S3 Fig — (A, B, C) Correlation plots between Bray-Curtis distance matrices calculated for bacteria within soil treatments, root, and shoot fractions. The R and p-values were calculated using Mantel tests. (A) Correlation plot of soil versus root. (B) Correlation plot of soil versus shoot. (C) Correlation plot of root versus shoot. (D, E, F) Correlation plots between Bray-Curtis distance matrices calculated for fungi within soil treatments, root, and shoot fractions. The R and p-values were calculated using Mantel tests. R and p-values colored in red were calculated excluding the cloud of large distances appearing in graphs panels D and E. (D) Correlation plot of soil versus root. (E) Correlation plot of soil versus shoot. (F) Correlation plot of root versus shoot. (TIF) [file pbio.3000534.s003.tif]

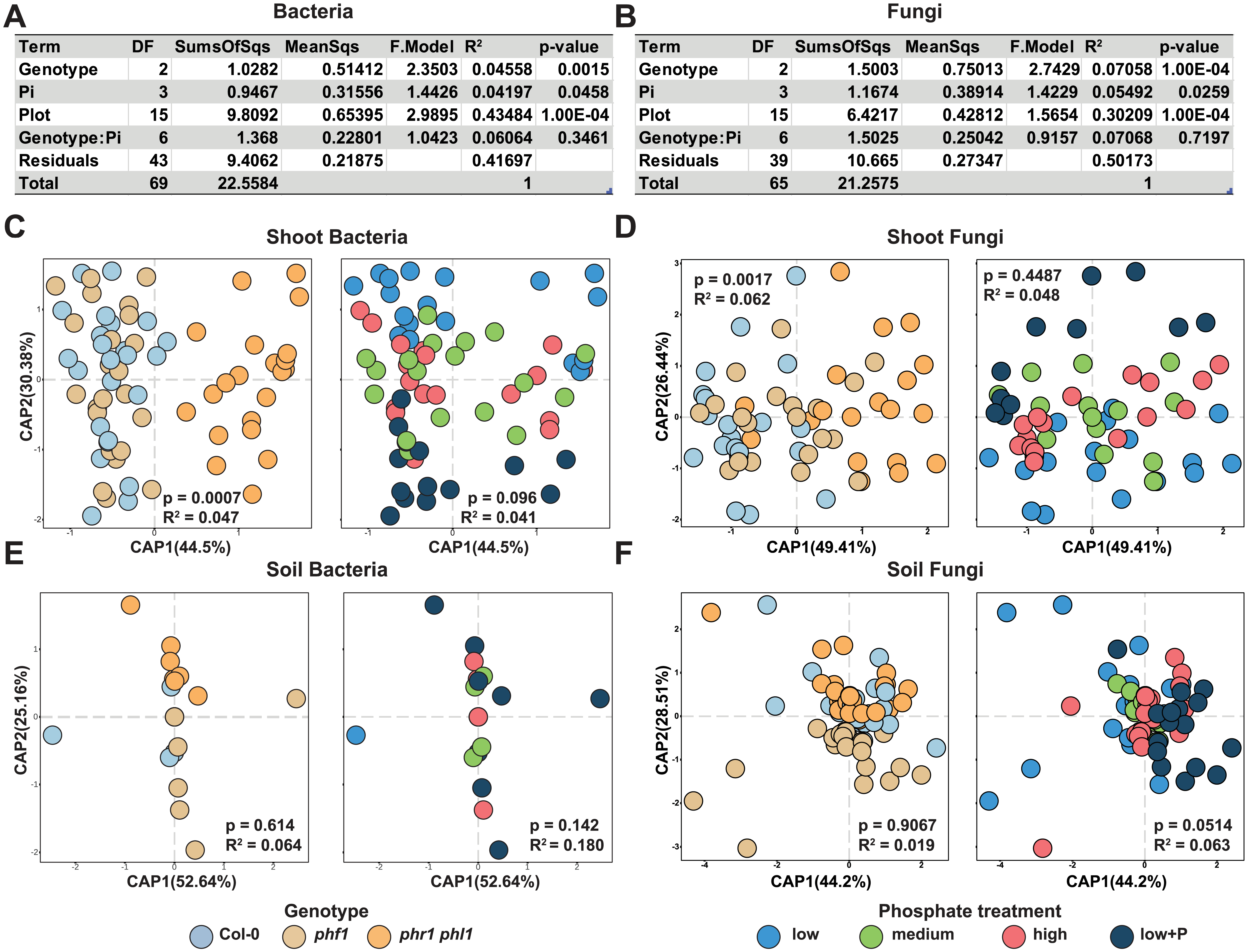

Supplement: S4 Fig — (A, B) PERMANOVA results showing the influence of the plant genotype and soil P concentration and their interaction on the assembly of the root (A) bacterial and (B) fungal communities. (C, D) CAP showing the effect of plant genotype and P content in the soil over the shoot (C) bacterial and (D) fungal communities. The p-value and R2 values in each plot are derived from a PERMANOVA model and correspond to the genotype and soil P term, respectively. (E, F) CAP showing the influence of genotype and P on the soil (E) bacterial and (F) fungal communities. Note smaller number of points in bacterial soil samples. The p-value and R2 values in each plot are derived from a PERMANOVA model and correspond to the genotype and soil P term, respectively. CAP, canonical analysis of principal coordinates; P, phosphorus; PERMANOVA, Permutational Multivariate Analysis of Variance. (TIF) [file pbio.3000534.s004.tif]

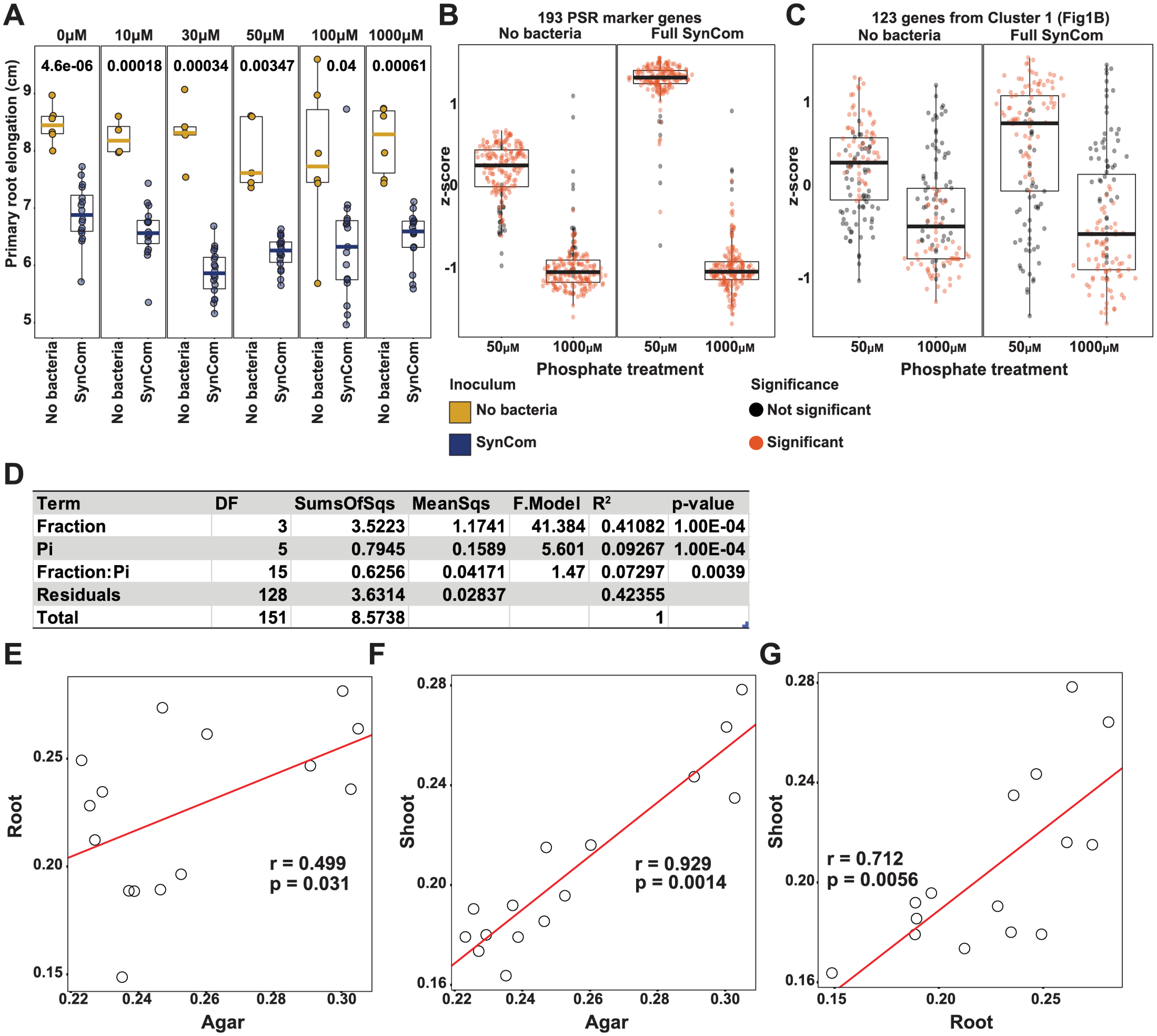

Supplement: S5 Fig — (A) Correlation heat map of the 13 edaphic factors reported in the work by Robbins and colleagues [43]. (B) Bar plot displaying the amount of variance in the edaphic factor matrix explained by PC. (C) Bar plot depicting the contribution of the different edaphic factors to the first 3 PC. Colors denote the direction of the variable in PCA space. (D) Proportion of the variance explained for the different variables in models including (M2) and excluding (M1) edaphic factors for bacteria (left) and fungi (right). Only variables with a statistically significant effect are shown. P, phosphorus; PC, principal component; PCA, principal component analysis. (TIF) [file pbio.3000534.s005.tif]

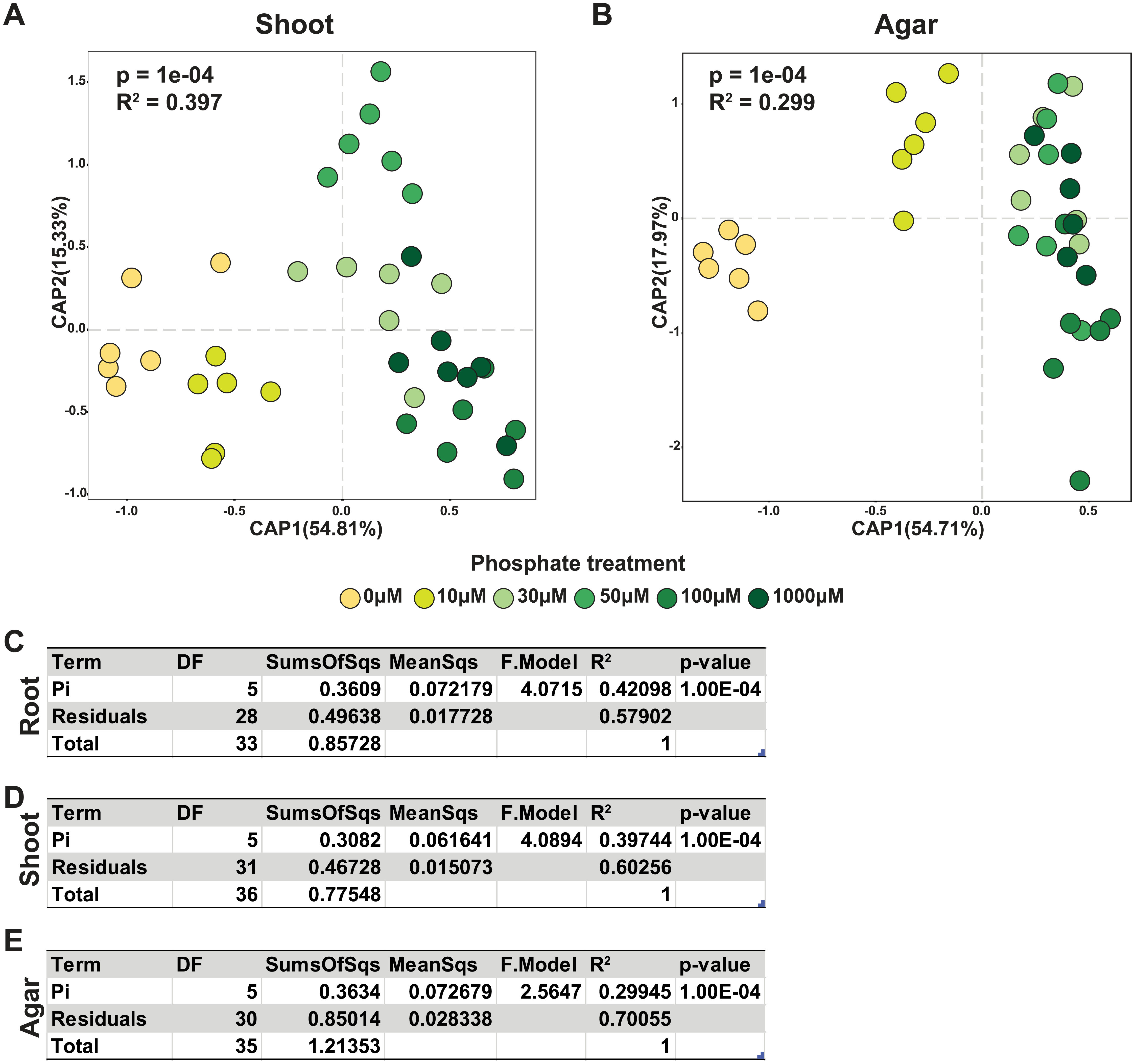

Supplement: S6 Fig — (A) Box plots displaying the primary root elongation of plants grown in a gradient of Pi concentrations in sterile conditions or with the SynCom. A t test was used for each Pi treatment to estimate differences between SynCom-treated and uninoculated plants. (B) Average expression of the 193 PSR markers genes in low (50 μM) and high (1,000 μM) Pi conditions within SynCom-treated and uninoculated plants. (C) Average expression of the 123 genes from Cluster 1 (Fig 1B) in low (50 μM) and high (1,000 μM) Pi conditions within SynCom-treated and uninoculated plants. (D) PERMANOVA model results showing the influence of the 2 variables (fraction and Pi concentration) and their interaction on the assembly of the bacterial community in the plant. (E, F, G) Correlation plots between Bray-Curtis distance matrices calculated for bacterial profile within agar, root, and shoot fractions. The R and p-values were calculated using Mantel tests. (E) Correlation plot of agar versus root. (F) Correlation plot of agar versus shoot. (G) Correlation plot of root versus shoot. PERMANOVA, Permutational Multivariate Analysis of Variance; Pi, orthophosphate; PSR, phosphate starvation response; SynCom, synthetic community. (TIF) [file pbio.3000534.s006.tif]

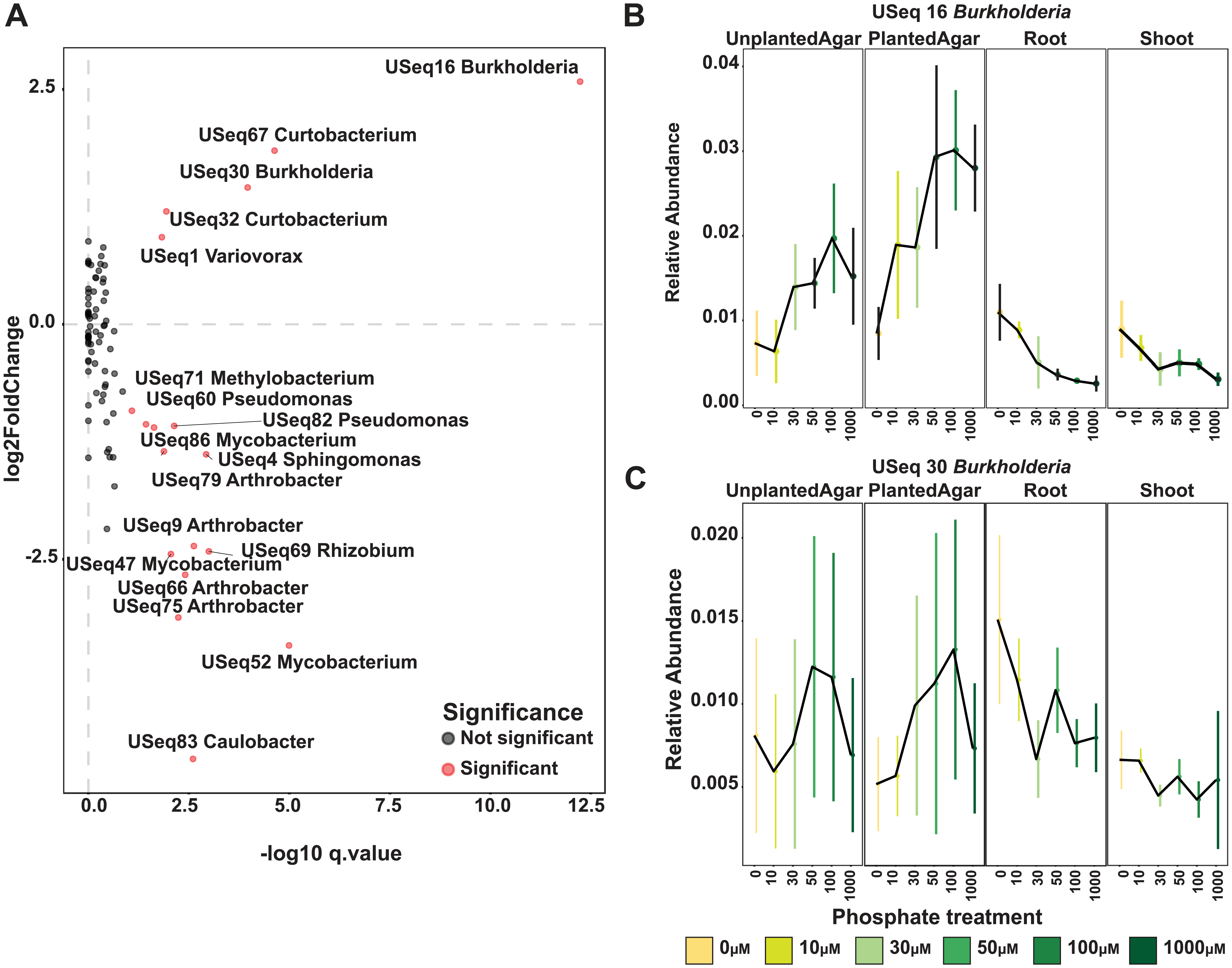

Supplement: S7 Fig — (A, B) CAP showing the influence of Pi concentration in the media on the bacterial communities in the (A) plant shoot and (B) agar. The bar graphs to the left of each plot depict the percentage of variability explained by statistically significant (p < 0.05) variables based on a PERMANOVA model. (C, D, E) PERMANOVA model results showing the influence of Pi concentration on the assembly of the bacterial community in (C) roots, (D) shoot, and (E) agar. CAP, canonical analysis of principal coordinates; PERMANOVA, Permutational Multivariate Analysis of Variance; Pi, orthophosphate. (TIF) [file pbio.3000534.s007.tif]

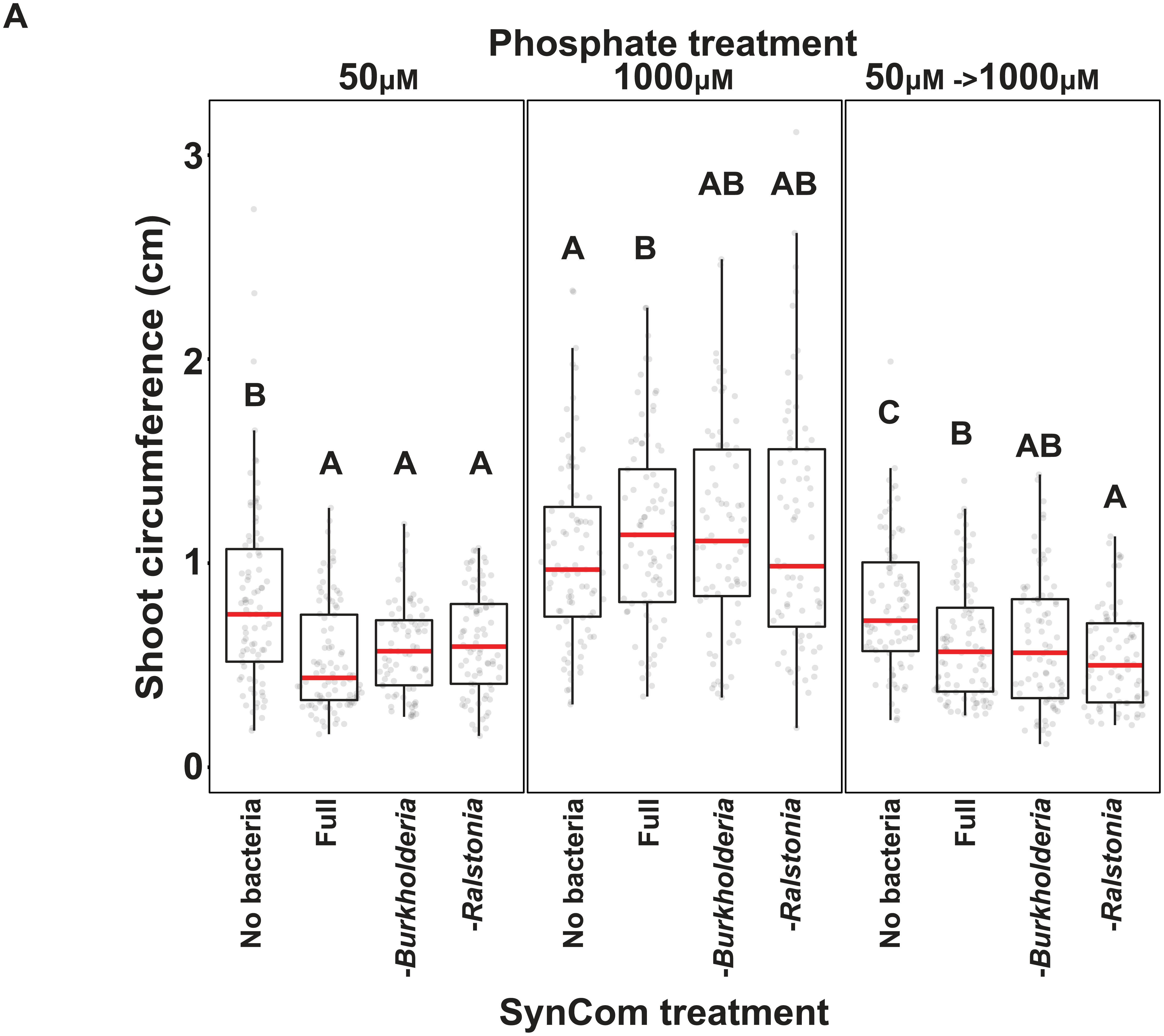

Supplement: S8 Fig — (A) Scatter plot (volcano plot) showing the results of the GLM interaction model between fraction and Pi concentration. The axes of the plot represent the output of the statistical test. The x-axis is the transformed q-value and the y-axis the log2 fold change. Each dot in the scatter plot represents a USeq. USeqs that showed a statistically significant fraction:Pi interaction are colored in red. USeqs genus and ID are displayed. The top right quadrant represents USeqs that are enriched in the plant tissues under low Pi conditions. (B, C) Relative abundance of Burkholderia Useqs 16 (B) and 30 (C) that exhibits a statistically significant (q < 0.1) Pi enrichment between the plant fractions and the agar fraction. The middle dot of each strip bar corresponds to the mean of that particular condition; the range of the strip bar corresponds to the 95% confidence interval of the mean. GLM, generalized linear model; Pi, orthophosphate; USeq, unique sequence. (TIF) [file pbio.3000534.s008.tif]

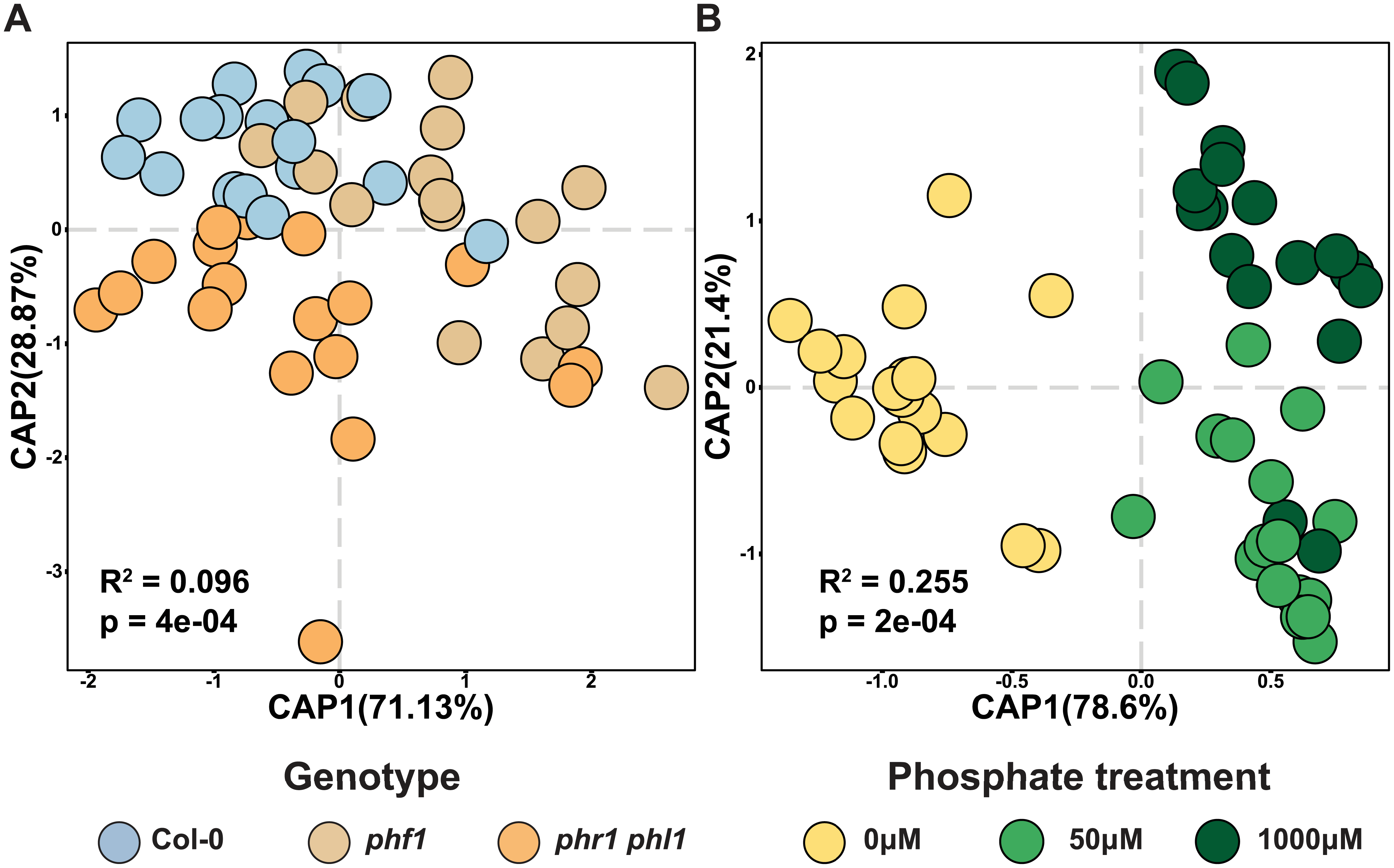

Supplement: S9 Fig — Box plots showing shoot circumference in plants exposed to different SynComs across 3 phosphate treatments. Statistically significant differences among SynCom treatments were computed within each phosphate treatment separately using an ANOVA model. Letters represent the results of the post hoc test. SynCom, synthetic community. (TIF) [file pbio.3000534.s009.tif]

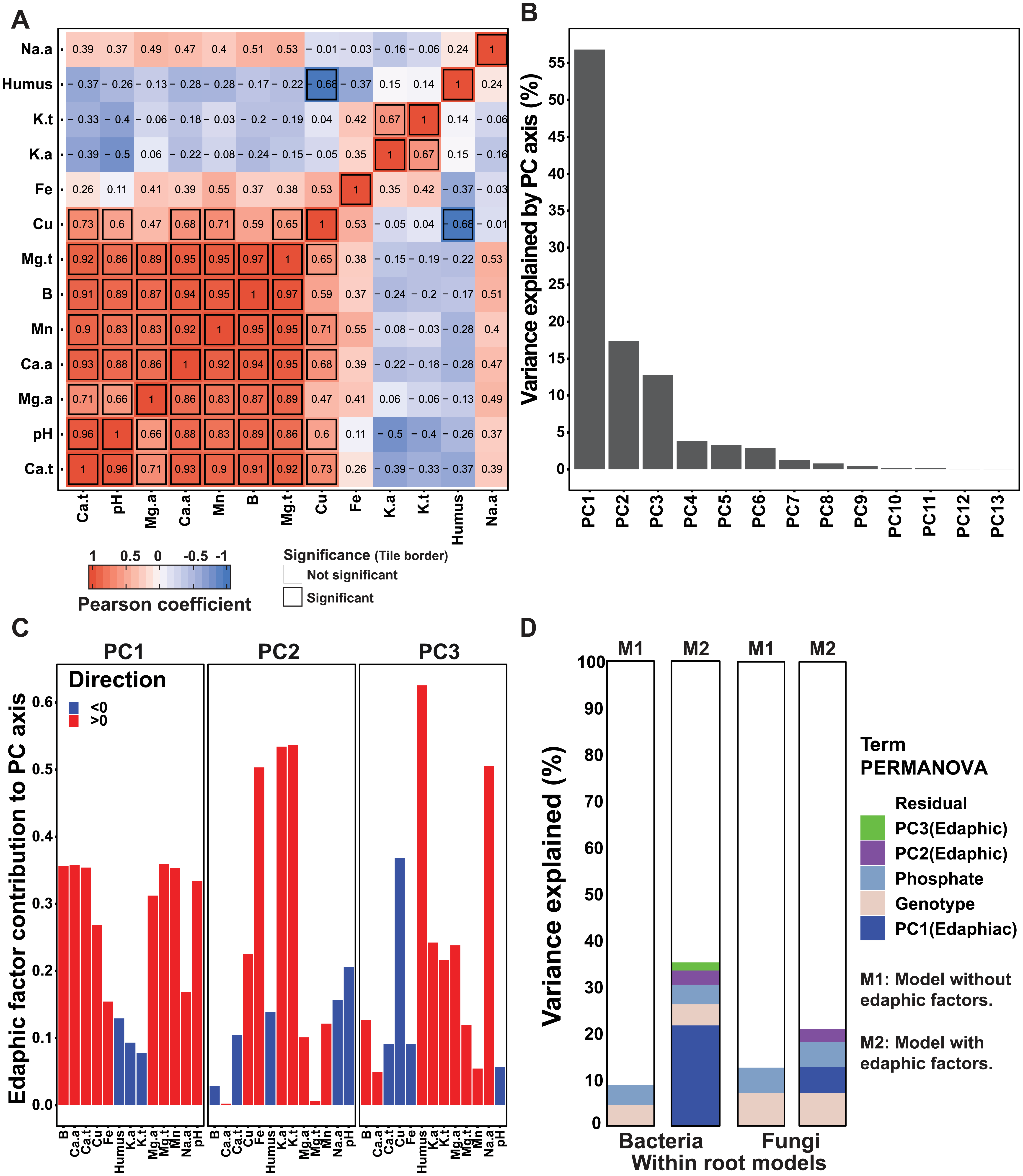

Supplement: S10 Fig — CAP showing the influence of plant genotypes (A) and agar Pi concentration (B) over the bacterial SynCom in the root. The p-value and R2 values inside each plot are derived from a PERMANOVA model and correspond to the genotype and Pi terms, respectively. CAP, canonical analysis of principal coordinates; PERMANOVA, Permutational Multivariate Analysis of Variance; Pi, orthophosphate; SynCom, synthetic community. (TIF) [file pbio.3000534.s010.tif]
